# Data-driven prediction of COVID-19 cases in Germany for decision making

**DOI:** 10.1186/s12874-022-01579-9

**Published:** 2022-04-20

**Authors:** Lukas Refisch, Fabian Lorenz, Torsten Riedlinger, Hannes Taubenböck, Martina Fischer, Linus Grabenhenrich, Martin Wolkewitz, Harald Binder, Clemens Kreutz

**Affiliations:** 1 Institute of Medical Biometry and Statistics, Faculty of Medicine and Medical Center, University of Freiburg, Stefan Meier Str. 26, Freiburg, 79104 Germany; 2 Institute of Physics, University of Freiburg, Hermann-Herder-Str. 3, Freiburg, 79104 Germany; 3 Centre for Integrative Biological Signalling Studies (CIBSS), Schänzlestr. 18, Freiburg, 79104 Germany; 4 German Aerospace Center, Earth Observation Center, Münchener Str. 20, Weßling, 82234 Germany; 5 Institute for Geography and Geology, Julius-Maximilians-Universität Würzburg, Am Hubland, Würzburg, 97074 Germany; 6 Robert-Koch-Institute, Department for Methodology and Research Infrastructure, Nordufer 20, Berlin, 13353 Germany; 7 Charité - Universitätsmedizin Berlin, Department of Dermatology, Venerology and Allergology, Luisenstraße 2, Berlin, 10117 Germany; 8 Freiburg Center for Data Analysis and Modelling (FDM), University of Freiburg, Ernst-Zermelo-Str. 1, Freiburg, 79104 Germany

**Keywords:** COVID-19, Infectious disease models, Input estimation, Ordinary differential equations, Parameter estimation, Nonlinear systems, SEIR models

## Abstract

**Background:**

The COVID-19 pandemic has led to a high interest in mathematical models describing and predicting the diverse aspects and implications of the virus outbreak. Model results represent an important part of the information base for the decision process on different administrative levels. The Robert-Koch-Institute (RKI) initiated a project whose main goal is to predict COVID-19-specific occupation of beds in intensive care units: *Steuerungs-Prognose von Intensivmedizinischen COVID-19 Kapazitäten* (SPoCK). The incidence of COVID-19 cases is a crucial predictor for this occupation.

**Methods:**

We developed a model based on ordinary differential equations for the COVID-19 spread with a time-dependent infection rate described by a spline. Furthermore, the model explicitly accounts for weekday-specific reporting and adjusts for reporting delay. The model is calibrated in a purely data-driven manner by a maximum likelihood approach. Uncertainties are evaluated using the profile likelihood method. The uncertainty about the appropriate modeling assumptions can be accounted for by including and merging results of different modelling approaches. The analysis uses data from Germany describing the COVID-19 spread from early 2020 until March 31st, 2021.

**Results:**

The model is calibrated based on incident cases on a daily basis and provides daily predictions of incident COVID-19 cases for the upcoming three weeks including uncertainty estimates for Germany and its subregions. Derived quantities such as cumulative counts and 7-day incidences with corresponding uncertainties can be computed. The estimation of the time-dependent infection rate leads to an estimated reproduction factor that is oscillating around one. Data-driven estimation of the dark figure purely from incident cases is not feasible.

**Conclusions:**

We successfully implemented a procedure to forecast near future COVID-19 incidences for diverse subregions in Germany which are made available to various decision makers via an interactive web application. Results of the incidence modeling are also used as a predictor for forecasting the need of intensive care units.

**Supplementary Information:**

The online version contains supplementary material available at (10.1186/s12874-022-01579-9).

## Background

The current COVID-19 pandemic is far from over and affects more or less every country on the globe. The evolution of new variants of concerns, such as Delta and possibly Omicron increase infectiousness of the disease around the globe. Several vaccines have been developed and came to widespread application in 2021 but did not yet reach enough people to effectively contain the virus evolution and spread.

In Germany, the situation in late fall of 2021 is grim: Hospitals and hospital personnel are working at their limit capacity to treat individuals infected with COVID-19. Due to exhausted capacities in some regions, the air force of the national army has started to fly patients across the country to enable treatment of every individual that needs intensive care, often including ventilation.

Mathematical models of infectious disease epidemiology have experienced a boost of attention since the beginning of the COVID-19 pandemic. One can divide these models into three categories according to their purpose: scenario simulation, nowcasting, and forecasting.

Scenario simulation focuses on different assumptions about some aspects of the model in order to compare and illustrate differences between several scenarios of in principle conceivable progressions of the transmission and other dynamics, which do not allow for proper uncertainty assessment. These approaches are used to examine the impact of changing certain parameters in the system, e.g. social behaviour, vaccination rate, etc, see e.g. [1]. Nowcasting focuses on the precise description of the present situation based on incomplete, noisy and/or systematically biased data about the current state [2, 3]. Forecasting tries to make predictions about the near future providing policy makers with reliable estimates of advancing developments [4]. Similar to nowcasting, forecasting is strongly oriented towards realistic settings. The work presented in this publication focuses on a near-future prediction and can therefore be classified as forecasting.

Resources of hospitals are limited and decision makers have to organize planning of capacities on a regional level. We provide a forecasting tool about the situation on the incidence level of cases as well as the intensive care unit occupation level.

### The SPoCK project

In Germany, local health authorities collect data about the infection dynamics on population level as mandated by the *Infektionsschutzgesetz “infection protection act”* (IfSG) and report it to the national public health institute, the *Robert Koch-Institut* (RKI). In addition, the *DIVI Intensivregister*, which is run by RKI with support of the *Deutschen Interdisziplinären Vereinigung für Intensiv- und Notfallmedizin “German Interdisciplinary Association for Intesive and Emergency Medicine”* (DIVI), collects and publishes data about the daily occupations of intensive care unit (ICU) capacities on the clinic level.

**Fig. 1:**
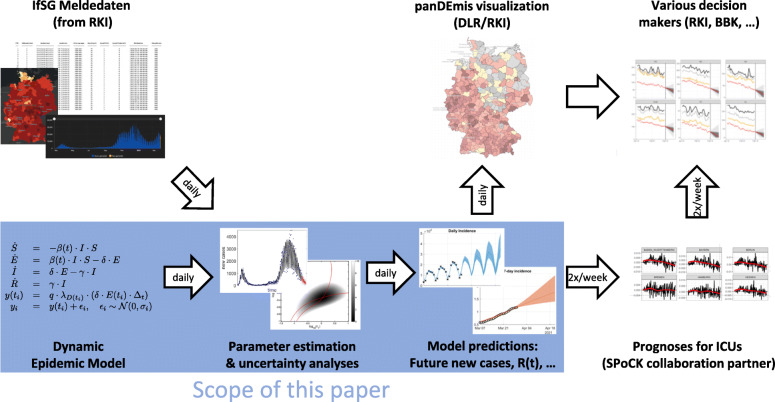
Schematic workflow of the SPoCK project. The SPoCK project predicts the needed hospital capacity of ICUs for COVID-19 patients. A key ingredient is the number of newly reported cases from the RKI which also has to be predicted (indicated by blue box). Results are used for visualization by the DLR and by decision makers, such as the BBK and RKI as well as local and regional health authorities

The project named *Steuerungs-Prognose von Intensivmedizinischen COVID-19 Kapazitäten* (SPoCK) makes use of these data sources and forecasts in a data-driven manner the number of occupied ICU beds. The workflow within the SPoCK project is depicted in Fig. 1.

Several decision makers including the *Bundesgesundheitsministerium “Federal Ministry of Health”* (BMG), the RKI, the local planners of ICU capacities as well as the *Bundesamt für Bevölkerungsschutz und Katastrophenhilfe “Federal Office of Civil Protection and Disaster Assistance”* (BBK) incorporate these predictions into their risk assessment of the current COVID-19 situation.

SPoCK is utilizing a two-step procedure:

1. Data-driven forecasting of the future number of daily infections with COVID-19. In addition, the predicted incidences are visualized on an interactive web application provided by the *Deutsches Luft- und Raumfahrtzentrum* (DLR) called *Pandemic Mapping and Information System for Germany* (panDEmis).
2. The number of occupied ICU beds is fitted and forecasted by our cooperation partners. The results of the first step are utilized as a main predictor to obtain short-term future predictions on the level of COVID-19-specific occupation of beds and hence ICU capacities.

In this paper, we describe the first step of Spock, i.e. fitting and short term forecasting of the newly reported cases of COVID-19 in Germany. That means, we describe the daily analysis and prediction and publication via panDEmis of incident cases of COVID-19 in different regions in Germany which are, in addition to the entire country, the 16 federal states (*Bundesländer*) and their 413 counties (*Land- und Stadtkreise*), summing to a total of 430 regions.

## Methods

A standard approach when describing infectious disease transmission are compartmental models or Susceptible-Infected-Recovered (SIR) -like models [5]. In general, both approaches divide the population into subpopulations with disjoint properties. Transition rates allow for flows between the subpopulations and define, in combination with the initial values of the subpopulations, the time evolution of the system. The ordinary differential equation (ODE) representation of the compartmental scheme we use is the well-known Susceptible-Exposed-Infected-Recovered (SEIR) model [6]:

1 $$\begin{array}{*{20}l}  \begin{array}{rrrrrl} \dot{S} &=& -\beta(t)\cdot I\cdot S/N & & \\ \dot{E} &=& \beta(t)\cdot I\cdot S/N & -\delta\cdot E & \\ \dot{I} &=& & \delta\cdot E & -\gamma\cdot I \\ \dot{R} &=& & & \gamma\cdot I \end{array} \end{array} $$

with *N*=*S*+*E*+*I*+*R* resembling the entire population and where the dot notation is used to indicate time derivatives. Furthermore *β*,*γ*,*δ* resemble the infection rate, the rate to become infectious and the rate with which one dies or recoveres, respectively. The rationale in choosing this model class is that it is concise which is important for frequent evaluation and allows for a more flexible infection time when compared with the standard SIR model.

A special characteristic of the current pandemic is the massive political and social reaction. In contrast to, e.g. the annual influenza season during which the social and professional life used to proceed pretty much as usual, the COVID-19 pandemic has led to vast political interventions and personal restrictions aiming mainly at the reduction of infections [7]. Within the SEIR scheme these changes over time can be described by a time-dependent infection rate *β*(*t*) which translates to an effective time-dependent reproduction number $R(t)=\frac {\beta (t)\cdot S}{\gamma \cdot N}$. The latter quantifies how many other people are infected on average by a single infectious individual and determines at which rate the number of currently infectious individuals is growing (*R*(*t*)>1) or decaying (*R*(*t*)<1). It should be noted that, despite the fact that *β*(*t*) is extrapolated as remaining constant (see Eq. 4), *R*(*t*) is not necessarily constant. This is because *R*(*t*) includes the monotonously decreasing susceptible density $\frac {S(t)}{N}$. The dynamics of all additional states can, for one example, be found in the supplement (Additional file 1).

There are several studies dealing with the problem of time-dependent infection rate in different manners. For example, at the beginning of the COVID-19 pandemic the impact of different non-pharmacological interventions (NPIs) was examined via step functions that implement *β*(*t*) via different variants of (smoothed) step functions, e.g. to examine the impact of different NPIs [8–11]. Often, these approaches are restricted to time ranges in which the infection rate is assumed to be constant or monotonously decreasing or increasing, respectively.

In contrast, we aim for a more general approach which enables the infection rate to vary flexibly, i.e. to decrease and/or increase repeatedly within the considered time range. This is necessary for an accurate description of the COVID-19 transmission dynamics since it is influenced by many factors that may vary over the course of the ongoing COVID-19 pandemics:

1. Various NPIs are implemented, repealed and reintroduced iteratively [12].
2. The population’s compliance to regulative measures changes over time [13].
3. Seasonal effects, e.g. weather conditions, lead to changes in infection risk [14].
4. Mutations alter the physiological mechanisms underlying the disease transmission and other aspects [15].
5. Vaccinations reduce the population’s susceptible fraction [16].
6. Air pollution may enhance COVID-19 severity [17].

Quantifying the effects of the above points on the infection rate is hardly feasible and within an evolving pandemic it is fairly impossible. Therefore, we omit an explicit formulation of the above effects and strive for an estimation of an effective infection rate. In order to fit a strictly positive and time-dependent infection rate simultaneously with the SEIR model’s parameters, we introduce the following parametrization for the infection rate:

2 $$\begin{array}{*{20}l} \beta(t) = b\cdot \frac{1}{1+\mathrm{e}^{-f(t)}}\:\:, \end{array} $$

where the argument of the exponential function is given by an interpolating cubic spline

3 $$\begin{array}{*{20}l}  f(t) = \text{cubic\_spline}\left(t,\{\tau_{i},u_{i}\}_{i\in\{1,\dots,n\}}\right)\:\:. \end{array} $$

We utilize joint estimation of input spline and ODE parameters as introduced for biological systems in [18]. The composition of the interpolating spline (3) with the logistic function (2) allows for a nearly arbitrary time dependence, while still ensuring that the infection rate *β*(*t*) is strictly positive, smooth and restricted to a maximal value *b*. The cubic spline curve is determined by estimated parameters *u*_*i*_=cubic_spline(*τ*_*i*_) that represent its values at fixed and evenly spaced dates *τ*_*i*_ for *i*∈{1, …, *n*−2} which cover the time range of observed data. We chose *n*=15 which leads to roughly one degree of freedom per month which turned out to be a reasonable choice during the development process. In general, there is a trade off: It should be flexible enough to describe all infection waves, but it is also necessary to have no overfitting in any of the fitted regions.

In our model, the last two spline knots are placed after the date *t*_Last_ of the last data point: *τ*_*n*−1_=*t*_Last_+50d and *τ*_*n*_=*t*_Last_+300d. The value *u*_*n*−1_ is fitted to allow for some flexibility in the most recent regime, whereas *u*_*n*_=0 is fixed for numerical stability and reflecting the end of the pandemic in at least 300 days.

The predictions for the infection dynamics are primarily determined by the time-dependent infection rate *β*(*t*). In general, assumptions for the future development of *β*(*t*) are difficult to justify as many different factors contribute to it. For illustrative purposes, several different assumptions could be made and visualised as done e.g. in various online simulator tools [19]. For example, one such scenario study nicely illustrates the effectiveness of a Test-Trace-Isolate strategy [20].

For a data-driven approach focused on short-term forecasts, we need to be more practical: For extrapolation purposes, we fix

4 $$ \beta(t>t_{\text{Last}}) = \beta(t_{\text{Last}})  $$

i.e. we assume the infection rate to be constant starting from the day where the last data point is reported. Alternatively, for *β*(*t*>*t*_Last_) some functional form incorporating the derivative or even higher-order derivatives could be utilized. As it is a priori totally unclear, which functional form and additional assumptions might be appropriate, we decided to go for the most simple ansatz by fixing it to *β*(*t*_Last_). Note also, that by fixing at *t*>*t*_Last_ we already have some kind of extending as the model system has an integrated delay due to its structure.

### Data-driven approach

Typically, there exist a multitude of model classes and structures which can be used to describe the same phenomenon. However, it is generally not possible to transfer results about estimated parameters between different models in a straightforward manner due to their differing mechanistic structures. To circumvent this problem, we here rely on a purely data-driven approach meaning that no prior knowledge about parameter values is incorporated into the optimization procedure. The only three a priori fixed parameters are the initial number of individuals in the susceptible, the exposed and the recovered state: *S*_init_,*E*_init_ and *R*_init_. Time point zero *t*_0_ is set to the first day that has at least a total of 100 reported cases to ensure the well-mixing assumption of ODE modeling. *S*_init_ was set to the total population of the respective region as given by the Federal Statistical Office of Germany [21]. *E*_init_ was set to *γ*·*I*_init_/*δ*, which is motivated by the assumption that $\dot {I}\approx 0$ at the beginning of an epidemic reflecting a slow onset. *R*_init_ is set to zero. The only remaining initial occupation number *I*_init_ is estimated from the data.

### Link between model and observed data

In order to calibrate the ODE model, it needs to be linked to the observed data. The data we use for calibration is the daily incidence *y*_*i*_ published by the reporting date (*Meldedatum*) *t*_*i*_ at the local health authority. Therefore, we introduce the observation function

5 $$\begin{array}{*{20}l} y(t_{i}) = q\cdot\lambda_{D(t_{i})}\cdot\left(\delta\cdot E(t_{i})\cdot \Delta\right)\:\:, \end{array} $$

where the parameters can be interpreted as follows:

- *q*∈[0,1] is the fraction of all infectious individuals that are detected and reported.
- *D*(*t*_*i*_)∈{1,...,7} is an index for the weekday at date *t*_*i*_ where {1,...,7} are naturally identified with the weekdays *W*={Monday,…,Sunday}.
- *λ*_*D*_ is a factor for the weekday *D* that adjusts for the weekly modulation occurring in the IfSG data (see Weekly modulation factors).
- (*δ*·*E*(*t*)·*Δ*) approximates the influx into the state *I*(*t*) of Eq. 1. As the considered data represents daily incidences, we set *Δ* to 1 day. This approximation of the true incidence quantity $\int _{t-1}^{t}\delta \cdot E(t^{\prime }) \mathrm {d}t^{\prime }$ is exact if the state *E*(*t*) remains constant within that day. Comparison with this exact but computationally much more expensive approach showed minor deviations for real data applications.

The observable function (5) connects the model’s predictions to the reported data. The observations are assumed to scatter around this mean according to a normal distribution:

6 $$\begin{array}{*{20}l}y_{i} = y(t_{i}) + \epsilon_{i},\quad\epsilon_{i}\sim\mathcal{N}(0,\sigma_{i}^{2})\:\:. \end{array} $$

As we are dealing with a count process we use the standard deviation inspired by a Poisson model

7 $$  \sigma_{i}=C\cdot\sqrt{1+y(t_{i})}\:\:  $$

where the addition of 1 accounts for numerical instabilities if the number of infected *y*(*t*_*i*_) becomes very low. As the standard deviation grows with the square root of the incidences, the variance grows linearly with the expectation value. The error parameter *C* is fitted jointly with all others.

#### Investigated time frame

The results of the presented ansatz are calculated on a daily basis. The data used for fitting consists then of a time course from the start of the pandemic, March 1st, 2020 through the most recent report with one data point per day. In this paper, we present the methodology and the results were generated on April 1st, 2021. The data fitted had therefore registered infections up to March 31st, 2021. We publish and assess predictions for a forecast horizon of three weeks. This period was selected because we think that the assumption of Eq. 4 is justifying no much longer time frame.

#### Weekly modulation factors

The IfSG data shows an oscillatory pattern with a period of one week which can be quickly evaluated by plotting distribution of incidences per weekday relative to the rolling 7-day average: we provide an analyzing figure in the supplement. The main reason for this is the reporting procedure, displaying a major delay during weekends, instead of actual infection dynamics. Therefore, we account for this effect within the observation function via seven weekday-specific factors *λ*_*D*_ with the integer *D*∈{1,...,7}. In order to

1. guarantee that the factors *λ*_*D*_ essentially do not change the 7-day-incidence and
2. separate the weekly modulation from a global scaling of the observation function, which is realized via the factor *q*,

we, furthermore, set the constraint that

8 $$\begin{array}{*{20}l} \sum_{D\in\{1,...,7\}}\lambda_{D}=7\:\:. \end{array} $$

As a consequence, we are left with six degrees of freedom to describe the weekly effects. For a convenient implementation in the used software, we introduce a Fourier series with six parameters *Θ*_weekly_={*A*_1_,*A*_2_,*A*_3_,*ϕ*_1_,*ϕ*_2_,*ϕ*_3_}:

9 $$\begin{array}{*{20}l}  \psi(t) = A_{0} + \sum_{k=1}^{3} A_{k}\cdot \cos\left(k\omega t + \phi_{k}\right) \end{array} $$

where offset and frequency are fixed to

10 $$\begin{array}{*{20}l} A_{0} = 1,\quad \omega = \frac{2\pi}{7\,\text{days}}\:\:. \end{array} $$

Instead of fitting the factors *λ*_*D*_ directly, we rewrite them in terms of equation (9) as

11 $$\begin{array}{*{20}l} \lambda_{D} = \frac{\psi(D)}{\sum_{j=1}^{7}\psi(j)}\:\: \end{array} $$

and calibrate the parameters *Θ*_weekly_. Doing so allows to set the amplitudes *A*_1_,*A*_2_ and *A*_3_ to zero in order to get an adjusted curve that does not feature the weekly oscillations and therefore reflects the ideal case of no reporting artifacts in the data.

#### Correction of last data points

The IfSG data published on date *t*_*n*_ contains information about the reported cases at all past dates *t*_*n*_,*t*_*n*−1_,…,*t*_1_ since the beginning of reporting. However, due to reporting delays between the test facilities, the local health authorities and the RKI, the data update from date *t*_*n*−1_ to *t*_*n*_ contains not only cases that were reported to the local health authorities at date *t*_*n*−1_, but also before that at dates *t*_*n*−2_,*t*_*n*−3_,… and so on. This means that the number of reported cases on day *t*_*n*_ will be underestimated especially for the most recent dates.

Meaningful handling of this data artifact can be done in at least two ways: For instance, one could choose to ignore some of the latest data points, since they are most prominently affected by this data artifact. An alternative is to estimate the systematic deviation from historically published data sets. In order to avoid the bias towards smaller incidences in the prediction, the data can be adjusted accordingly. Therefore, one assumes, that the future data sets of *t*_*n*_ will not change reported counts older than four weeks *t*_*n*−28_. Let $N_{t_{1}}^{t_{2}}$ denote the number of reported cases, that were published at time point *t*_1_ to be reportedly infected at date *t*_2_ where $N_{t_{1}}^{t_{2}>t_{1}} = 0$ as future cases cannot be reported. Then, one can learn from this history of published data sets the correction factor *CF*_*k*_

12 $$  CF_{k} = \frac{\sum_{\hat t} N_{\hat t}^{\hat t-k}}{\sum_{\hat t} N_{t_{\text{Last}}}^{\hat t-k}}  $$

the initial publication of *k* day old counts had to be corrected to obtain the number in the latest data set *t*_*n*_. The factors *CF*_*k*_ can then be applied to the newest data set.

This was done for Germany and all the federal states separately. We showcase the resulting differences of these two data preprocessing strategies in Averaging of approaches section. We give some summary statistics of this quantity in the supplement.

For the county level, this adjustment is not as crucial for two reasons: 1) the count numbers are much lower, so the stochasticity can lead to wrong correction factors and 2) the shape of the estimated dynamics is inherited from the federal states in our model.

### Parameter estimation

In general, we follow the maximum likelihood estimation (MLE) approach. As there are a total of 429 regions for which the data has to be fitted and predictions are calculated, we rely on a two-step procedure to reduce computation time which is described in the following paragraphs.

#### Federal states and Germany

The parameter estimation problem given by the above defined ODE model and the IfSG daily incidence data is solved separately for Germany and each federal state by an MLE approach. The latter has been well established for ODE models [22]. The deviation between data and the model’s observation function as specified in Eq. 5 is minimized, taking into account the error model of Eqs. 6 and (7). The simultaneous parameter estimation of the spline parameters *u*_*i*_ follows the lines of [18]. In particular, no explicit regularization term is implemented that penalizes non-vanishing spline curvatures. A full list of parameters *Θ* and their estimation results $\hat \Theta $ is shown in the supplement (Additional file 1) for one example, the region of Germany.

#### County level

Analysis at the rural and urban county level (*Land- and Stadtkreise*) is important to obtain a spatially resolved picture of the infection dynamics in Germany. The previously described approach is computationally not feasible because the analysis of 429 regions cannot be performed within 24 hours without access to a sufficiently large computing cluster which can be used 24/7 without queuing. Moreover, the number of infected individuals can generally be so small at the county level that inference and prediction based on a purely deterministic model is not appropriate. Therefore, we used the results on the higher-level administrative structure, i.e. the fitted model of the federal state, as prior information about the dynamics, and scaled it down to the county level for predictions.

More specifically, the county-level data was used to merely estimate two parameters in a county-specific manner: the scaling parameter *q* from equation (5), which in this context can be related to the proportion of current infections occurring in the county *c*, and the error parameter *C* from equation (7) which quantifies the stochasticity of county-level observations analogous to its meaning on the level of federal states. All other parameter values for a county *c* are taken from the estimated set of parameters $\hat \Theta _{FS(c)}$ for the corresponding federal state *FS*(*c*).

The county-level dynamics might change rapidly as new clusters of infection emerge. For predictions, it is important that such rapid changes are detected by the model calibration procedure, i.e. fitting of *q* and *C* has to account for such rapid changes. We implemented this requirement by exponentially weighting down the county level data observed in the past by increasing the standard deviations via

13 $$ {}\sigma^{2}_{i}\longleftarrow\frac{\sigma^{2}_{i}}{w_{i}}\:\:,\quad w_{i}=A\cdot\sqrt{\left(\exp{(t_{i}-t_{\text{Last}})/\tau}\right)^{2}+\left(w_{\min}/A\right)^{2}}\:\:.  $$

Here, *A*=7.56 denotes the normalization factor that ensures that the sum of all weights *w*_*i*_ is equal to one. Furthermore, *w*_min_=0.01·*A* denotes the minimal weight factor used for data observed in the past. *w*_min_ is necessary for numerical reasons: the first summand of the square root is exponentially decreasing towards zero and would (without additional second summand) lead to a divergence of the used standard deviation. The value of 0.01 is somewhat arbitrary. It effectively serves as a lower bound on the weights (or upper bound on standard deviation, respectively) for data points that are long time ago. Thorough evaluation of this hyperparameter of value 0.01 has not been performed, however it is not expected to have a crucial impact on results. Moreover, we chose *τ*=7 as time-constant of this weighting step. To be clear, on the county-level, *σ*_*i*_ from equation (7) should be thought of as first being transformed according to the mapping (13) before entering equation (6) as the standard deviation of Gaussian observation errors.

Just as the analysis for the federal states, the described scaling procedure for the counties is updated on a daily basis, i.e. the county-specific parameters *q* and *C* are updated every day. This accounts for time-dependent deviations of the local infection history on the federal state level, i.e. each county has an individual kinetics.

### Calculation of uncertainties

To quantify the uncertainty in the predictions of the model, our forecasting tool provides confidence intervals along with proposed predictions. Here, we describe two main sources of uncertainties: parameter uncertainty and approach uncertainty. The first is captured by simulating all parameter combinations that agree with the observed data as will be explained in Profile likelihood analysissection, the second is incorporated by running the analysis with several models as detailed in Averaging of approaches section.

#### Profile likelihood analysis

For non-linear models, uncertainties for estimated parameters can be determined using the *profile likelihood* (PL) method which estimates parameter values that still ensure agreement of model and data to a certain confidence level in a pointwise and iterative manner [23]. This approach has been showcased for infectious disease models [24]. Parameter uncertainties naturally translate to prediction uncertainties which can be analyzed systematically [25]. Following the given references, we simulate the data-compatible parameter combinations from the parameter profiles and then take the envelope of the resulting family of curves to obtain confidence intervals.

One could also analyze the uncertainty of a model prediction directly via the *prediction profile likelihood* method [26]. Prediction profiles need to be computed via a costly iterative fitting procedure for each predicted quantity and time point separately. However, by using the parameter combinations from the profile likelihood method, we can calculate uncertainties for any desired model quantities and time points only by simulation, thus rendering this method more efficient for our purposes.

#### Averaging of approaches

When utilizing ODE models to describe certain aspects of reality, a multitude of assumptions are implicitly made, which include (but are not limited to) the selected model structure, the noise model of the data, the appropriate data preprocessing. All these decisions result in a certain *approach*. These necessary decisions along the modeling process impact the space of possibly described and therefore also predicted dynamics. To account for this origin of uncertainty, we perform the procedure described so far simultaneously for several approaches and merge their results into one comprehensive result. The latter is done by taking the mean / minimum / maximum of the different approaches’ MLE / lower bound / upper bound curves. Accounting for different modeling decisions prevents overconfidence in the results.

## Results

Since April 2020, the described methodology has delivered daily predictions and the *ansatz* has evolved and several changes and refinements have been implemented. Currently, the resulting predictions for ICU bed capacity, which use estimated incidences derived by the present paper as a main predictor, are reported two times per week to public health decision makers. The presented methodology and results were generated on April 1st, 2021. The data fitted had therefore registered infections up to March 31st, 2021.

### COVID-19 spread in Germany

**Fig. 2:**
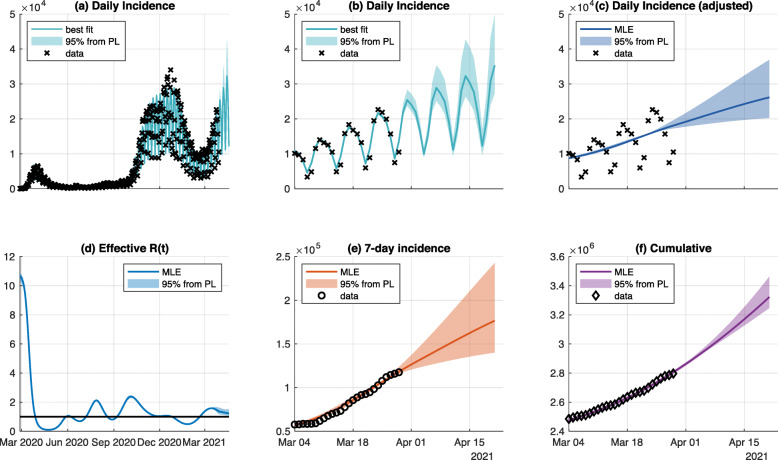
Fit and prediction for Germany. The incidence data of the entire time course is fitted (panel **a**) to estimate all dynamic parameters including the time-dependent infection rate that corresponds to *R*(*t*) (panel d). Predictions of incidences (panels **b** and **c**) and derived quantities (panels **e** and **f**) for a zoomed in time span are shown. 95%-confidence intervals (color-shaded areas) are inferred by profile likelihood calculation. The independent results for all federal states are shown in the supplement (Additional file 1)

For the aggregated data over all of Germany, we obtained a fit and predictions with uncertainties as shown in Fig. 2. The fitted data can be described by the model (panels a and b) and the prediction is a reasonable continuation of the last data points. Since we adjusted for weekday effects, the adjusted trajectory can be assessed and results in a smoothing of the trajectory (panel c). The estimated reproduction number *R*(*t*) oscillates around a value of 1 and illustrates the effect of politics’ countermeasures and the population’s compliance to them (panel d). In general, oscillations in dynamical systems often are attributed to a feedback with delay, which is also the case here for the reproduction number *R*(*t*). Several additional quantities of interest, such as the 7-day incidence (panel e) or the cumulative number of cases (panel f) can be computed from the model’s predictions. In addition, the associated confidence intervals of these quantities can be determined using the parameter sets below the 95% threshold of likelihood profiles. We stress here again, that only the incidence data was used for model calibration (panels a and b).

### COVID-19 spread in subregions of Germany

**Fig. 3:**
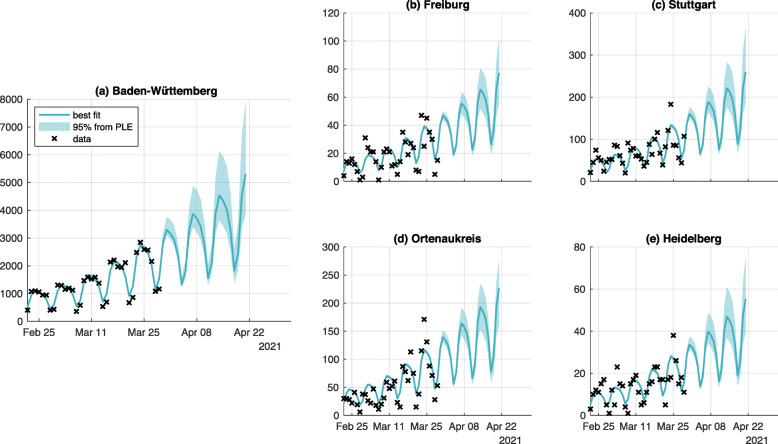
Fit and prediction for one federal state and four counties. The dynamic of the one exemplary federal state Baden-Württemberg (panel **a**) governs the dynamics of the corresponding Landkreise, four of them are shown here (panels **b** through **e**). For regions with fewer inhabitants, lower case numbers are expected: note the different scaling of the y-axis for federal state and counties

For the county-level (*Landkreise*) we obtain results by the scaling approach described in County level section. The shape of dynamics is preserved and describes the latest data. Due the exponential scaling on later data points, it is unlikely that the entire time course is described well by the scaled dynamics. As we are primarily interested in the forecast, we display only the latest time interval. The data is more noisy due lower numbers of cases and inhabitants (Fig. 3). Here, we show already merged results for clarity (see Approach averaging section). Results of all the counties can be found in the supplement (Additional file 1), where we also display already merged results for clarity (see Approach averaging section).

### Approach averaging

**Fig. 4:**
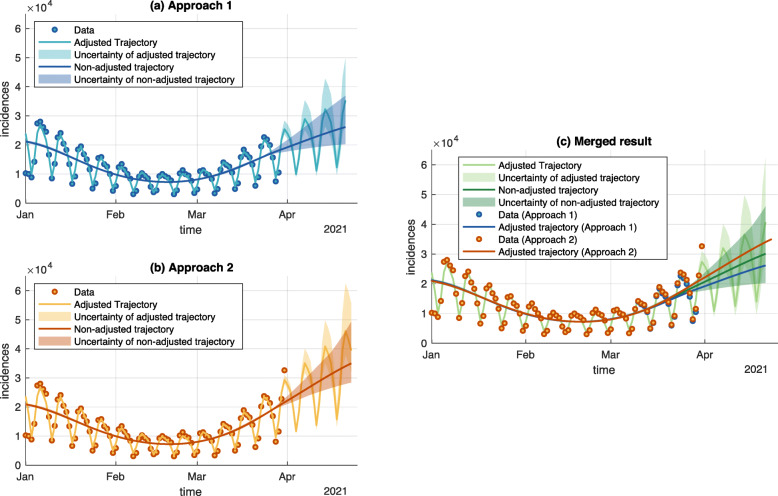
Merged Approaches for the example of Germany. The two approaches differ in their data handling strategies for considering reporting delays: Approach 1 (panel **a**) simply ignores the two latest data points. Approach 2, in contrast, uses estimated correction factors on the latest data points (panel **b**). The result of the merging (panel **c**) indicates that both approaches describe the data well, but make differing predictions. Therefore the resulting uncertainty is bigger than the individual uncertainties. In general, this procedure generalizes to more different approaches

The analyses can be carried out for different approaches representing a variety of a priori equally feasible modeling strategies. To account for the uncertainty that arises from (possibly over-)simplifying modelling assumptions, those different approaches are analyzed independent from each other. After results for all regional entities, i.e. federal states (as in Fig. 2 and counties Fig. 3) have been obtained for each approach, the results are merged into one comprehensive prediction, which features by construction (see Averaging of approaches) a higher uncertainty, now including both the uncertainty in the data and the uncertainty which modeling strategy is used. We illustrate this for two different approaches which differ only in the handling of the most recent data points (Fig. 4). In general, this methodology generalizes to an arbitrary number of different approaches with the available computing resources as the only limiting factor.

### Availability of results

**Fig. 5:**
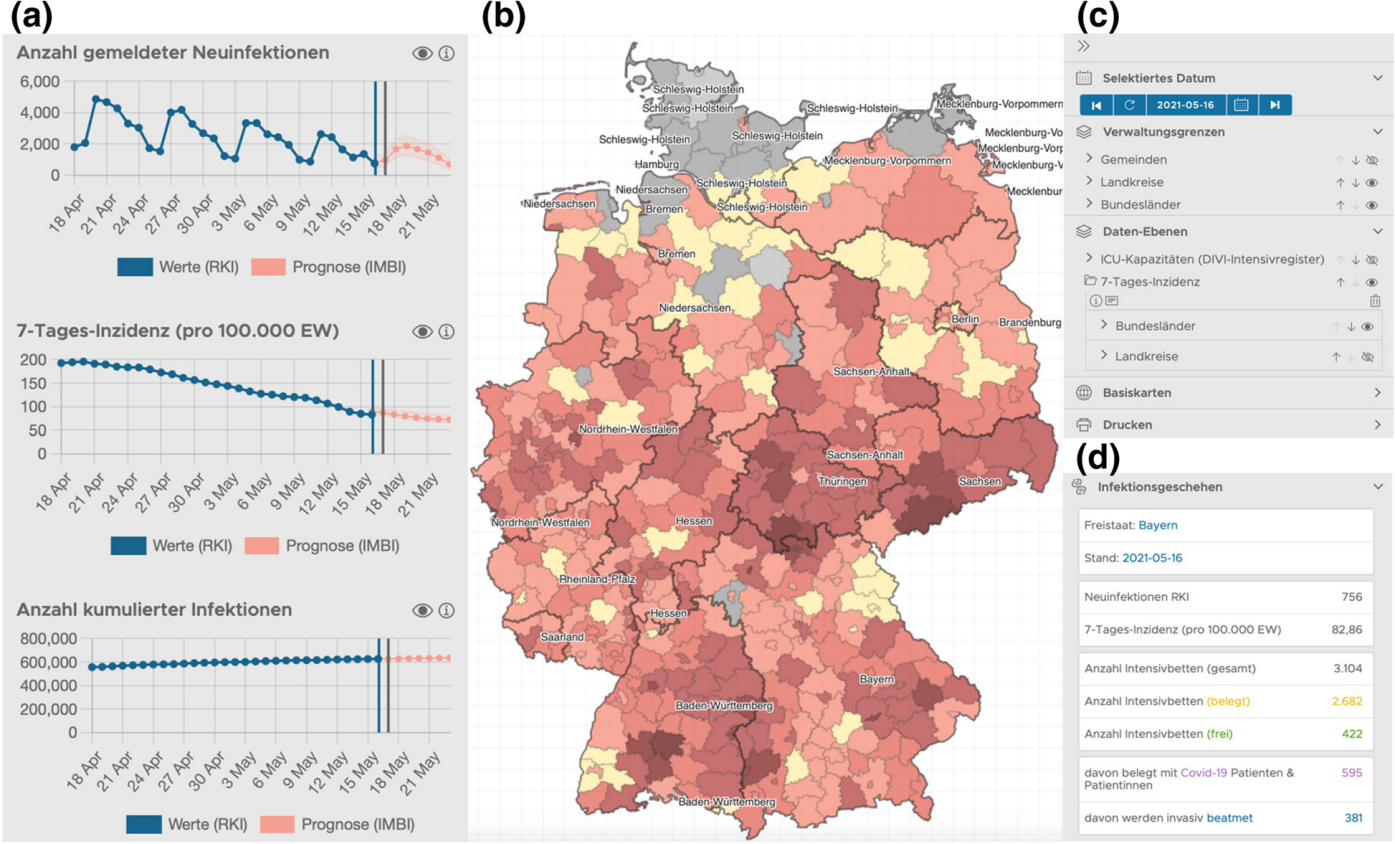
panDEmis visualization. On the interactive web application called panDEmis, predictions for incidences, 7-day average, as well as cumulative cases can be inspected for all subregions (panel **a**). The region can be selected through a map indicating all the regions (panel **b**). For the chosen regional district, historic data sets and predictions can be selected and different layers can be chosen for visualization (panel **c**). Additionally, key figures about the current pandemic situation, such as incidences and ICU bed capacities are displayed for the selected region (panel **d**)

Sound political or social decisions are based on an empirical or prognostic foundation. To make the daily generated predictions available to various stakeholders, the forecasts are integrated into a web-application called panDEmis: In this interactive application, the recent infection situation is analyzed and displayed. For all registered users of the *DIVI Intensivregister* the tool is available at https://pandemis.dlr.de/de/#/overview. Current capacities of hospital beds and intensive care units, exposed population in the catchment areas of hospitals are merged with the forecast data. The combined display of all available data sets allows a situation picture for each day including also for past and future time steps. Figure 5 shows different features of the web-application from May 17th, 2021 for the occurrence of infection in the map entire Germany (panel b), as well as for the selected administrative district of Bayern (panel a). Here, the blue graphs represent 1) the daily reported new infections by RKI, 2) the incidence of COVID-19 cases in the past 7 days per 100,000 people and 3) the cumulative infections. The prognosis is displayed as red curve, including a 95% confidence interval. All data can be interactively analyzed and visualized for different administrative units, i.e. federal states and county level.

The results of this incidence modeling approach are also a main predictor for a prediction analysis of ICU beds. The results of this second analysis step which is not detailed within this paper, is available for all registered users of the *DIVI Intensivregister* at https://www.intensivregister.de/#/aktuelle-lage/prognosen.

## Discussion

Different model classes as ODE models or stochastic differential equation (SDE) models with or without mixed effects could be used for a data-driven parameter estimation approach. An SDE approach might be beneficial for small regions with low infection numbers or during times with very low total infection numbers. In these cases local outbreaks dominate the infection dynamics and the population is not *well-mixed* which renders an ODE approach ineffective. A well-mixed system (or here: population) implies that the infection probability for all susceptible persons is equally high or low and infection dynamics follows some averaged infection probability. For the presented regional entities, the underlying assumptions for ODE modeling are reasonable and the ODE model was successfully adapted. We here focused on a pragmatic procedure that allows daily analysis and reliably calculates predictions.

When fitting data about the number of reported cases of an infectious disease outbreak, it is beneficial to fit incidences (or fluxes) instead of the total (or cumulative) number of cases [27]. The residuals of a fit on cumulative data will be correlated by construction as every data point must be higher than the previous one, which clearly conflicts wit the following: Most noise models assume independent measurement errors. Thus, the uncertainty will be underestimated in these cases and obtained results will be overly confident. By fitting the model to incidence data, the measurement errors are not correlated by this effect. Of course, there can be additional reasons for correlations in the residuals. A good example for this is the prominent weekday effect in the data: If it was not corrected for in the observation function, this effect would lead to correlated residuals.

The presented modeling approach heavily relies on the time-dependent infection rate *β*(*t*). We assume dynamic processes to be continuously differentiable which leads to a smoothing of possible steps in the real infection rate which might occur due to rapid policy changes. Also, the temporal change *β*(*t*) incorporates many different mechanisms, which include but are not limited to: vaccinations, NPIs, changes in compliance to NPIs, viral mutations, seasonality and testing frequency. For an assumed constant vaccination rate, we saw that our approach delivers the same results when omitting the explicit vaccination state since *β*(*t*) is flexible enough to compensate the vaccination effect. The time dependence of *β* leads to an oscillation of reproduction number *R*(*t*). This is in line with several publications [11, 28, 29] reporting similar behavior of the reproduction number.

In general, it is a priori unclear how much flexibility this function should have. In the presented procedure, this corresponds to the number of knots employed in the spline. The spline’s freedom should allow for a good fit of the dynamics, but also prevent overfitting.

Furthermore, the dynamics of the prediction are primarily determined by the value of *R*(*t*) at the latest data point. Hence, this value should not be estimated by too few data points meaning that the last spline knot should not be too close to the end of the time series.

Any prediction model used for forecasting should not exceed a certain time period as the future infection rate is hard to determine. But even at a short prediction time span, it is unclear how recent political measures and the population’s resulting behavior will alter the future infection rate. Therefore, we assume *β*(*t*) to be constant starting at the last data points. By additional precise knowledge about the effect of planned or recently made political decisions or other effects like weather conditions, this assumption could be further refined.

In contrast to other modeling approaches, we do not feed the actual NPIs into the model, but can instead correlate the estimated time development in a second step of the infection rate to NPIs. Quantifying the NPIs’ effect and time lag on *R*(*t*) is difficult as most NPIs are not imposed or lifted independently of each other and estimates will therefore be highly correlated [30]. This means, our modeling *ansatz* cannot contribute to the quantification of the NPIs’ effect on infection numbers. Similarly, age- and time-resolved contact patterns did not enter our modeling *ansatz* and we can therefore not infer any quantitative statement regarding these quantities. Our main focus was predictions of case numbers and there are (by construction) no reliable estimates of future NPIs and/or contact patterns.

Whenever discussing the required amount of flexibility to obtain a good model fit, one should be aware of biasvariance-tradeoff: The introduction of more parameters included to explain a certain time dependence (reducing the bias), the bigger the resulting prediction uncertainty will be (increasing the variance). Similar arguments can be made when discussing the amount of utilized spline parameters or accounting for age structure. More available and consistent data can help.

There are no explicit states in our model to distinguish between recovered and dead people, mainly for the reason that there is no reliable data over the entire time course for those quantities. Recovered individuals are not tested to be non-sick anymore, and people who died were not consistently assessed in real-time in Germany. These omission from the model make quantitative assessments of death rates, (probably time dependent) risk of death and recovery rates not possible. As the goal was to predict development of case numbers, and these events happen downstream without a feedback, these shortcomings are not crucial to us.

Furthermore, the unobserved infected and infectious individuals are not in an explicit state. This fact is compensated by two aspects: Firstly, the used data does not contain information about the duration from beginning of infectivity to reporting to the local health authority. Thus, since the additional state would not help to better describe the used data, it is omitted. Secondly, the factor *q* introduced in the observation function in Link between model and observed data section accounts for individuals that are overseen at all times. The estimated dark figure from Eq. 5 when fitting only incidence data is in the presented modeling approach in most regions compatible with a broad set of values ranging from 0.1 to 1 within the confidence level. This means that anywhere between 10% to 100% of all cases are detected by local authorities and both edge cases still agree sufficiently with the data. Therefore, the dark figure can not be estimated solely based on reported incidence cases. For reliable determination of the dark figure, additional testing in pre-specified cohorts is necessary.

## Conclusions

We presented a data-driven ODE approach to fit and predict incidences of COVID-19 cases for different subregions of Germany. The key ingredients in doing so are 1) likelihood-based estimation and uncertainty quantification and 2) a time-dependent infection rate which is estimated by utilizing a cubic spline. All parameters are estimated from data and uncertainty in parameter estimates are translated to prediction uncertainty. As many different modeling assumptions will affect the outcomes, we average over similarly plausible approaches to account for this source of uncertainty. A major constraint for a feasible analysis strategy is a maximum runtime of 24 hours as the analysis should be repeated on a daily basis in an automated manner including the respectively newest data set.

In the future, more work for validation of competing modeling approaches and comparison of the various efforts undertaken in the currently highly dynamic field of mathematical modeling of infectious diseases is needed and will certainly be seen.

## Supplementary Information

**Additional file 1** We provide a supplement to give more insights about utilized models and obtained results.

## Data Availability

Data is collected and published to the public by Robert-Koch-Institute on a daily basis. The datasets used and/or analysed during the current study are available from the corresponding author on reasonable request. Results from modelling analyses comprise big data sets and require specialized software to interpret: Matlab with Data2Dynamics [31]. The source code used during the current study are available from the corresponding author on reasonable request.

## References

[1] Malkov E (2020). Simulation of coronavirus disease 2019 (COVID-19) scenarios with possibility of reinfection. Chaos Solitons Fractals.

[2] an der Heiden M, Hamouda O (2020). Erfassung der SARS-CoV-2-Testzahlen in Deutschland - Nowcasting. Epidemiologisches Bull.

[3] Günther F, Bender A, Katz K, Küchenhoff H, Höhle M (2020). Nowcasting the COVID-19 pandemic in bavaria. Biom J.

[4] Shinde GR, Kalamkar AB, Mahalle PN, Dey N, Chaki J, Hassanien AE (2020). Forecasting Models for Coronavirus Disease (COVID-19): A Survey of the State-of-the-Art. SN Comput Sci.

[5] Kermack WO, McKendrick AG (1927). A contribution to the mathematical theory of epidemics. Proc R Soc A.

[6] Keeling MJ, Rohani P (2008). Modeling Infectious Diseases in Humans and Animals.

[7] Maier BF, Brockmann D (2020). Effective containment explains subexponential growth in recent confirmed COVID-19 cases in China. Science.

[8] Dehning J, Zierenberg J, Spitzner FP, Wibral M, Neto JP, Wilczek M, Priesemann V. Inferring change points in the spread of COVID-19 reveals the effectiveness of interventions. Science. 2020; 369(6500). 10.1126/science.abb9789. 10.1126/science.abb9789 PMC7239331 32414780

[9] Linka K, Peirlinck M, Kuhl E (2020). The reproduction number of COVID-19 and its correlation with public health interventions. Comput Mech.

[10] Flaxman S, Mishra S, Gandy A, Unwin HJT, Mellan TA, Coupland H, Whittaker C, Zhu H, Berah T, Eaton JW, Monod M, Ghani AC, Donnelly CA, Riley S, Vollmer MAC, Ferguson NM, Okell LC, Bhatt S (2020). Estimating the effects of non-pharmaceutical interventions on COVID-19 in Europe. Nature.

[11] Dings C, Götz K, Och K, Sihinevich I, Selzer D, Werthner Q, Kovar L, Marok F, Schräpel C, Fuhr L, Türk D, Britz H, Smola S, Volk T, Kreuer S, Rissland J, Lehr T. Mathematische Modellierung und Vorhersage von COVID-19 Fällen,Hospitalisierung (inkl. Intensivstation und Beatmung) und Todesfällen in dendeutschen Bundesländern. 2021. https://covid-simulator.com/wp-content/uploads/2021/04/Report_2021_03_31.pdf. Accessed 1 Apr 2021.

[12] Mendez-Brito A, El Bcheraoui C, Pozo-Martin F (2021). Systematic review of empirical studies comparing the effectiveness of non-pharmaceutical interventions against COVID-19. J Infect.

[13] WHO Regional Office for Europe (2020). Pandemic fatigue – reinvigorating the public to prevent COVID-19. Policy framework for supporting pandemic prevention and management.

[14] Fontal A, Bouma MJ, San-José A, López L, Pascual M, Rodó X (2021). Climatic signatures in the different COVID-19 pandemic waves across both hemispheres. Nat Comput Sci.

[15] Ramesh S, Govindarajulu M, Parise RS, Neel L, Shankar T, Patel S, Lowery P, Smith F, Dhanasekaran M, Moore T (2021). Emerging SARS-CoV-2 Variants: A Review of Its Mutations, Its Implications and Vaccine Efficacy. Vaccines.

[16] Harder T, Külper-Schiek W, Reda S, Treskova-Schwarzbach M, Koch J, Vygen-Bonnet S, Wichmann O. Effectiveness of COVID-19 vaccines against SARS-CoV-2 infection with the Delta (B.1.617.2) variant: second interim results of a living systematic review and meta-analysis, 1 January to 25 August 2021. Euro Surveill Bull Eur Sur Les Mal Transmissibles Eur Commun Dis Bull. 2021;26(41). 10.2807/1560-7917.ES.2021.26.41.2100920. 10.2807/1560-7917.ES.2021.26.41.2100920 PMC8518304 34651577

[17] Ali N, Fariha KA, Islam F, Mishu MA, Mohanto NC, Hosen MJ, Hossain K (2021). Exposure to air pollution and COVID-19 severity: A review of current insights, management, and challenges. Integr Environ Assess Manag.

[18] Schelker M, Raue A, Timmer J, Kreutz C (2012). Comprehensive estimation of input signals and dynamics in biochemical reaction networks. Bioinformatics.

[19] Noll NB, Aksamentov I, Druelle V, Badenhorst A, Ronzani B, Jefferies G, Albert J, Neher RA. COVID-19 Scenarios: an interactive tool to explore the spread and associated morbidity and mortality of SARS-CoV-2. medRxiv. 2020;2020–050520091363. 10.1101/2020.05.05.20091363.

[20] Contreras S, Dehning J, Loidolt M, Zierenberg J, Spitzner FP, Urrea-Quintero JH, Mohr SB, Wilczek M, Wibral M, Priesemann V (2021). The challenges of containing SARS-CoV-2 via test-trace-and-isolate. Nat Commun.

[21] Kreisfreie Städte und Landkreise nach Fläche, Bevölkerung und Bevölkerungsdichte am 31.12.2019 - Statistisches Bundesamt. https://www.destatis.de/DE/Themen/Laender-Regionen/Regionales/Gemeindeverzeichnis/Administrativ/04-kreise.html. Accessed 1 Oct 2021.

[22] Raue A, Schilling M, Bachmann J, Matteson A, Schelker M, Kaschek D, Hug S, Kreutz C, Harms BD, Theis FJ, Klingmüller U, Timmer J (2013). Lessons Learned from Quantitative Dynamical Modeling in Systems Biology. PLoS ONE.

[23] Kreutz C, Raue A, Kaschek D, Timmer J (2013). Profile likelihood in systems biology. FEBS J.

[24] Tönsing C, Timmer J, Kreutz C. Profile likelihood-based analyses of infectious disease models. Stat Methods Med Res. 2017;962280217746444. 10.1177/0962280217746444. 10.1177/0962280217746444 29512437

[25] Steiert B, Raue A, Timmer J, Kreutz C (2012). Experimental Design for Parameter Estimation of Gene Regulatory Networks. PLoS ONE.

[26] Kreutz C, Raue A, Timmer J (2012). Likelihood based observability analysis and confidence intervals for predictions of dynamic models. BMC Syst Biol.

[27] King AA, Domenech de Cellès M, Magpantay FMG, Rohani P. Avoidable errors in the modelling of outbreaks of emerging pathogens, with special reference to Ebola. Proc R Soc B Biol Sci. 2015;282(1806). 10.1098/rspb.2015.0347. 10.1098/rspb.2015.0347 PMC4426634 25833863

[28] Khailaie S, Mitra T, Bandyopadhyay A, Schips M, Mascheroni P, Vanella P, Lange B, Binder SC, Meyer-Hermann M (2021). Development of the reproduction number from coronavirus SARS-CoV-2 case data in Germany and implications for political measures. BMC Med.

[29] Abbott S, Hellewell J, Thompson RN, Sherratt K, Gibbs HP, Bosse NI, Munday JD, Meakin S, Doughty EL, Chun JY, Chan Y-WD, Finger F, Campbell P, Endo A, Pearson CAB, Gimma A, Russell T, Flasche S, Kucharski AJ, Eggo RM, Funk S, CMMID COVID modelling group (2020). Estimating the time-varying reproduction number of SARS-CoV-2 using national and subnational case counts. Wellcome Open Res.

[30] Haug N, Geyrhofer L, Londei A, Dervic E, Desvars-Larrive A, Loreto V, Pinior B, Thurner S, Klimek P (2020). Ranking the effectiveness of worldwide COVID-19 government interventions. Nat Hum Behav.

[31] Raue A, Steiert B, Schelker M, Kreutz C, Maiwald T, Hass H, Vanlier J, Tönsing C, Adlung L, Engesser R, Mader W, Heinemann T, Hasenauer J, Schilling M, Höfer T, Klipp E, Theis F, Klingmüller U, Schöberl B, Timmer J (2015). Data2Dynamics: a modeling environment tailored to parameter estimation in dynamical systems. Bioinformatics.

