# Supplementary material for: Data-driven prediction of COVID-19 cases in Germany for decision making
Source: BMC Med Res Methodol. 2022 Apr 20;22:116. doi: 10.1186/s12874-022-01579-9 (PMC9019290; doi:10.1186/s12874-022-01579-9)
Supplement: Supplementary file 1 — Additional file 1 We provide a supplement to give more insights about utilized models and obtained results. [file 12874_2022_1579_MOESM1_ESM.pdf]

Supplementary material to

Data-Driven Prediction of COVID-19 Cases in Germany for Decision Making

Lukas Refisch, Fabian Lorenz, Torsten Riedlinger,
Hannes Taubenböck, Martina Fischer, Linus Grabenhenrich,
Martin Wolkewitz, Harald Binder and Clemens Kreutz

Contents

1 Modelling choices **2**

2 Weekday effect in data **3**

3 Correction Factor **4**

4 Model equations: SIR model with a spline input **5**
4.1 Dynamic variables . . . 5
4.2 Input variables . . . 5
4.3 Reactions . . . 6
4.4 ODE system . . . 6
4.5 Derived variables . . . 7
4.6 Conditions . . . 7
4.7 Observables . . . 8

5 Comprehensive results for one region: Germany **9**
5.1 Model variables and trajectories . . . 9
5.2 Fit to data . . . 10
5.3 Parameter estimation and confidence intervals . . . 11

6 Results for federal states **14**
6.1 Predictions for all federal states . . . 14
6.2 Weekly factor . . . 23

7 Results for all regions and counties: Landkreise **24**

1 Modelling choices

Table 1: **Necessary considerations for dynamical modeling**
For an ODE-model description of current Sars-Cov-2 pandemic at least the following points should be taken into account. This list is most probably not complete.

**Topic**	**What did we use**	**Possible Alternative(s)**	**Reasoning**
Data source	RKI "Meldedaten"	JHU, ECDC, Zeit Online ...	official German agency for reporting infectious disease cases
Quantity to fit	daily incidence	cumulative data	cumulative cases numbers are not independent of each other, so: uncertainty would be underestimated
Time point zero	day with >100 total cases	first day of reported infection	stochasticity at the beginning cannot be described with ODE approach.
Age structure	not taken into account	fit age groups of RKI data set	Number of parameters grows at least quadratically, but amount of data linearly when adding age groups. Also, computation time becomes an issue
Vaccination	not taken into account	include info on vaccinations	For constant vaccnation rate, beta(t) incorporates vaccination effect. No information on county level available.
Reporting delay	ignoring latest two days OR adjusting by historical data	ignoring it altogether	latest data points affect prediction, hence important.
Infection rate β(t)	cubic spline with 15 d.o.f	piecewise constant function between different NPIs	many unknown effects altering infection rate
Weekly oscillation	is accounted for	assuming no weekly data artifact	latest data points affect prediction, hence important.
Mutations	not taken into account	use additional sequencing data for fitting model	no comprehensive data on mutations available for Germany

2 Weekday effect in data

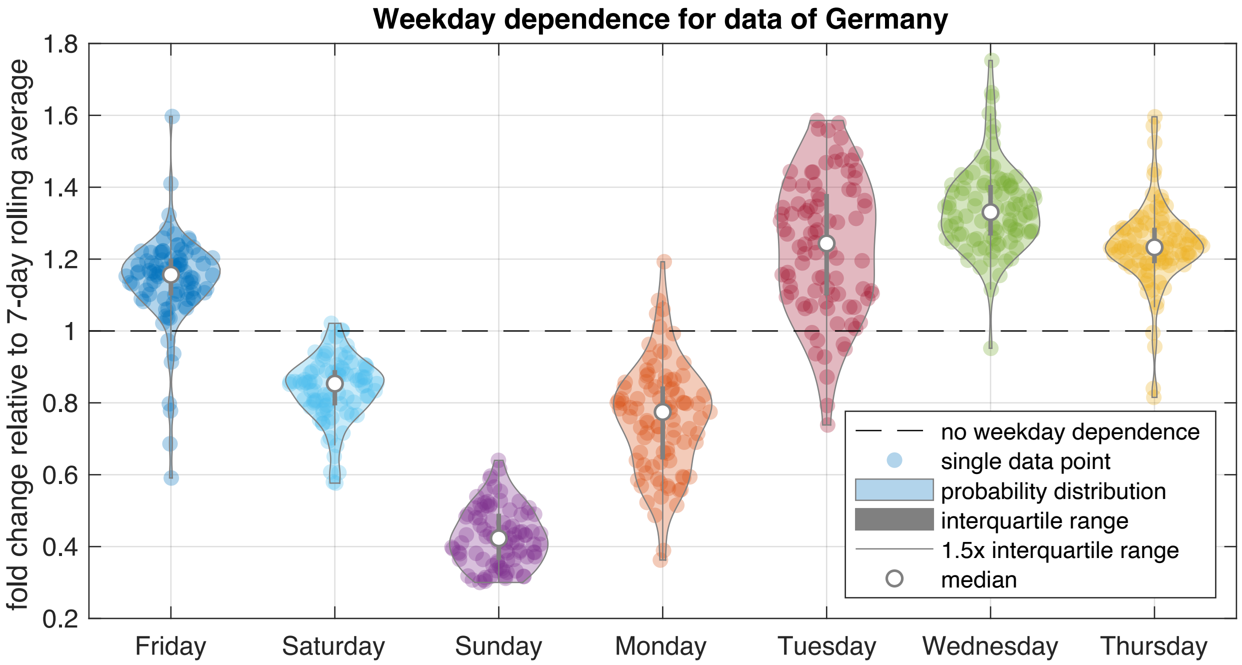

Figure 1: **Weekday has effect on reporting probability**
In the data, there is a clear dependence on the weekday. Plotted is the distribution of the fraction (or fold change) of the daily value and the 7-day-rolling average for that day per weekday. If there was no dependence on the weekday in the data, then these fractions should fluctuate around the value of one (dashed black line).
Note, that all quantity in this figure were computed based on the published incidence data - no ODE modeling has been performed for this. The time course from March 2020 through 1st December 2021 was analyzed for this particular figure.

3 Correction Factor

Table 2: **Summary statistics of correction factor**
For every federal state and Germany we calculated correction factors. The correction factor accounts for delayed reporting and is calculated from historic data sets. Here, we display summary statistics depending on number of days that have passed for the day that shall be corrected. Ideally, this value is 1 which corresponds to no correction at all.
The table can be read as follows: In the latest data set, the number of incident cases that has been reported for the day before yesterday (delay of two days) needs - on average - to be multiplied by a factor of 1.26 (or increased by 26%). For individual federal states this number might differ. A measure of how much it varies across regions is the standard deviation given in column 3.

**delay in days**	**mean** (CF)	**standard deviation** (CF)
1	2.13	0.70
2	1.26	0.28
3	1.12	0.09
4	1.09	0.05
5	1.09	0.04
6	1.08	0.03
7	1.06	0.02
8	1.05	0.02
9	1.04	0.02
10	1.04	0.02
...	...	...

4 Model equations: SIR model with a spline input

We present here the full list of equations of the model used for fitting time course data of federal states and Germany.

4.1 Dynamic variables

The model contains 4 dynamic variables. The dynamics of those variables evolve according to a system of ordinary differential equations (ODE) as will be defined in the following. The following list indicates the unique variable names and their initial conditions.

- **Dynamic variable 1:** Susceptibles

$$[\mathrm{Susceptibles}](\mathrm{t}=0) = \mathrm{init\_Susceptibles}$$

- **Dynamic variable 2:** Exposed

$$[\mathrm{Exposed}](\mathrm{t}=0) = \mathrm{init\_Exposed}$$

- **Dynamic variable 3:** Infectious

$$[\mathrm{Infectious}](\mathrm{t}=0) = \mathrm{init\_Infectious}$$

- **Dynamic variable 4:** Removed

$$[\mathrm{Removed}](\mathrm{t}=0) = 0$$

4.2 Input variables

The model contains 3 external inputs variables. Those variables evolve according to a regular algebraic equation. They are calculated before the ODE systems is solved and can appear in reaction rate equations. The following list indicates the unique variable names and their corresponding equations.

- **Input variable 1:** InputSpline

$$\begin{aligned}[\mathrm{InputSpline}](\mathrm{t}) = \mathrm{spline\_15}\Big(&\mathrm{t}, 0, 5, \frac{1}{13}\cdot \mathrm{tLast}, \mathrm{spValue2}, \frac{2}{13}\cdot \mathrm{tLast}, \mathrm{spValue3}, \frac{3}{13}\cdot \mathrm{tLast}, \mathrm{spValue4},\\ &\frac{4}{13}\cdot \mathrm{tLast}, \mathrm{spValue5}, \frac{5}{13}\cdot \mathrm{tLast}, \mathrm{spValue6}, \frac{6}{13}\cdot \mathrm{tLast}, \mathrm{spValue7},\\ &\frac{7}{13}\cdot \mathrm{tLast}, \mathrm{spValue8}, \frac{8}{13}\cdot \mathrm{tLast}, \mathrm{spValue9}, \frac{9}{13}\cdot \mathrm{tLast}, \mathrm{spValue10},\\ &\frac{10}{13}\cdot \mathrm{tLast}, \mathrm{spValue11}, \frac{11}{13}\cdot \mathrm{tLast}, \mathrm{spValue12}, \frac{12}{13}\cdot \mathrm{tLast}, \mathrm{spValue13},\\ &\mathrm{tLast}+50, \mathrm{spValue14}, \mathrm{tLast}+300, 5, 0, 0\Big)\end{aligned}$$

  which is the notation of the modelling tool for a spline with 13 fitted values on an equally spaced time axis fixed future nodes. See here for documentation of input splines.

- **Input variable 2:** InputSplineEnd

$$[\mathrm{InputSplineEnd}](\mathrm{t}) = [\mathrm{InputSpline}](\mathrm{t}=\mathrm{tLast})$$

- **Input variable 3:** fixSpline

$$[\mathrm{fixSpline}](\mathrm{t}) = \mathrm{step\_1}\left(\mathrm{t}, 0, \mathrm{tLast}, 1\right)$$

4.3 Reactions

The model contains 3 reactions. Reactions define interactions between dynamics variables and build up the ODE systems. The following list indicates the reaction laws and their corresponding reaction rate equations. In the reaction rate equations dynamic and input variables are indicated by square brackets. The remaining variables are model parameters that remain constant over time.

- **Reaction 1:**

$$\text{Susceptibles} \longrightarrow \text{Exposed}$$

$$v_1 = -\frac{[\text{Infectious}] \cdot [\text{Susceptibles}] \cdot \text{b} \cdot \left(\frac{[\text{fixSpline}]-1}{\text{e}^{-[\text{InputSpline}]}+1} - \frac{[\text{fixSpline}]}{\text{e}^{-[\text{InputSplineEnd}]}+1}\right)}{[\text{Exposed}] + [\text{Infectious}] + [\text{Removed}] + [\text{Susceptibles}]}$$

- **Reaction 2:**

$$\text{Exposed} \longrightarrow \text{Infectious}$$

$$v_2 = [\text{Exposed}] \cdot \text{rate\_infectious}$$

- **Reaction 3:**

$$\text{Infectious} \longrightarrow \text{Removed}$$

$$v_3 = [\text{Infectious}] \cdot \text{rate\_removal}$$

4.4 ODE system

The specified reation laws and rate equations v determine an ODE system. The time evolution of the dynamical variables is calculated by solving this equation system.

$$\begin{aligned} \text{d[Susceptibles]/dt} &= -v_1 \\ \text{d[Exposed]/dt} &= +v_1 - v_2 \\ \text{d[Infectious]/dt} &= +v_2 - v_3 \\ \text{d[Removed]/dt} &= +v_3 \end{aligned}$$

Substituting the reaction rates v_i yields:

$$\text{d[Susceptibles]/dt} = -\frac{[\text{Infectious}] \cdot [\text{Susceptibles}] \cdot \text{b} \cdot [\text{b\_time\_dependence}]}{[\text{Total\_population}]}$$

$$\text{d[Exposed]/dt} = \frac{[\text{Infectious}] \cdot [\text{Susceptibles}] \cdot \text{b} \cdot [\text{b\_time\_dependence}]}{[\text{Total\_population}]} - [\text{Exposed}] \cdot \text{rate\_infectious}$$

$$\text{d[Infectious]/dt} = [\text{Exposed}] \cdot \text{rate\_infectious} - [\text{Infectious}] \cdot \text{rate\_removal}$$

$$\text{d[Removed]/dt} = [\text{Infectious}] \cdot \text{rate\_removal}$$

The ODE system was solved by a parallelized implementation of the CVODES algorithm. It also supplies the parameter sensitivities utilized for parameter estimation.

4.5 Derived variables

The model contains 6 derived variables. Derived variables are calculated after the ODE system was solved. Dynamic and input variables are indicated by square brackets. The remaining variables are model parameters that remain constant over time.

- **Derived variable 1:** b_time_dependence

$$[\mathrm{b\_time\_dependence}](t) = \frac{[\mathrm{fixSpline}]}{e^{-[\mathrm{InputSplineEnd}]} + 1} - \frac{[\mathrm{fixSpline}] - 1}{e^{-[\mathrm{InputSpline}]} + 1}$$

- **Derived variable 2:** Total_confirmed

$$[\mathrm{Total\_confirmed}](t) = [\mathrm{Infectious}] + [\mathrm{Removed}]$$

- **Derived variable 3:** Total_population

$$[\mathrm{Total\_population}](t) = [\mathrm{Exposed}] + [\mathrm{Infectious}] + [\mathrm{Removed}] + [\mathrm{Susceptibles}]$$

- **Derived variable 4:** R_zero_t

$$[\mathrm{R\_zero\_t}](t) = -\frac{b \cdot \left(\frac{[\mathrm{fixSpline}]-1}{e^{-[\mathrm{InputSpline}]}+1} - \frac{[\mathrm{fixSpline}]}{e^{-[\mathrm{InputSplineEnd}]}+1}\right)}{\mathrm{rate\_removal}}$$

- **Derived variable 5:** R_t

$$[\mathrm{R\_t}](t) = -\frac{[\mathrm{Susceptibles}] \cdot b \cdot \left(\frac{[\mathrm{fixSpline}]-1}{e^{-[\mathrm{InputSpline}]}+1} - \frac{[\mathrm{fixSpline}]}{e^{-[\mathrm{InputSplineEnd}]}+1}\right)}{\mathrm{rate\_removal} \cdot ([\mathrm{Exposed}] + [\mathrm{Infectious}] + [\mathrm{Removed}] + [\mathrm{Susceptibles}])}$$

- **Derived variable 6:** Total_confirmed_flux

$$[\mathrm{Total\_confirmed\_flux}](t) = [\mathrm{Exposed}] \cdot \mathrm{rate\_infectious}$$

4.6 Conditions

Conditions modify the model according to replacement rules. New model parameters can be introduced or relations between existing model parameters can be implemented. The following list are default conditions that can be replace my experiment specific conditions defined separately for each data set.

$$\begin{aligned}
b &\rightarrow \mathrm{R_null} \cdot \mathrm{rate_removal} \\
\mathrm{init_Exposed} &\rightarrow \frac{\mathrm{init_Infectious} \cdot \mathrm{rate_removal}}{\mathrm{rate_infectious}} \\
\mathrm{init_Exposed} &\rightarrow \frac{\mathrm{init_Infectious_Germany} \cdot \mathrm{rate_removal}}{\mathrm{rate_infectious}} \\
\mathrm{init_Infectious} &\rightarrow \mathrm{init_Infectious_Germany} \\
\mathrm{init_Susceptibles} &\rightarrow 83186719
\end{aligned}$$

The value of init_Susceptibles is the number of inhabitants of Germany and will obviously differ dependent on the fitted federal state.

4.7 Observables

The following observable and its error function are added to fit the data. The weekday modulation factor λ_D is described in the main paper.

- **Observable:** Germany_confirmed_flux

$$\text{Germany\_confirmed\_flux(t)} = [\text{Total\_confirmed\_flux}] \cdot \text{scaleCon} \cdot \lambda_D$$
$$\sigma\{\text{Germany\_confirmed\_flux}\}(\text{t}) = \text{sigma} \cdot \sqrt{[\text{Total\_confirmed\_flux}] \cdot \text{scaleCon} + 1}$$

Note, that for scaling down the dynamics from federal state level to county level, the error function is adjusted by equation (13) of the main manuscript.

5 Comprehensive results for one region: Germany

5.1 Model variables and trajectories

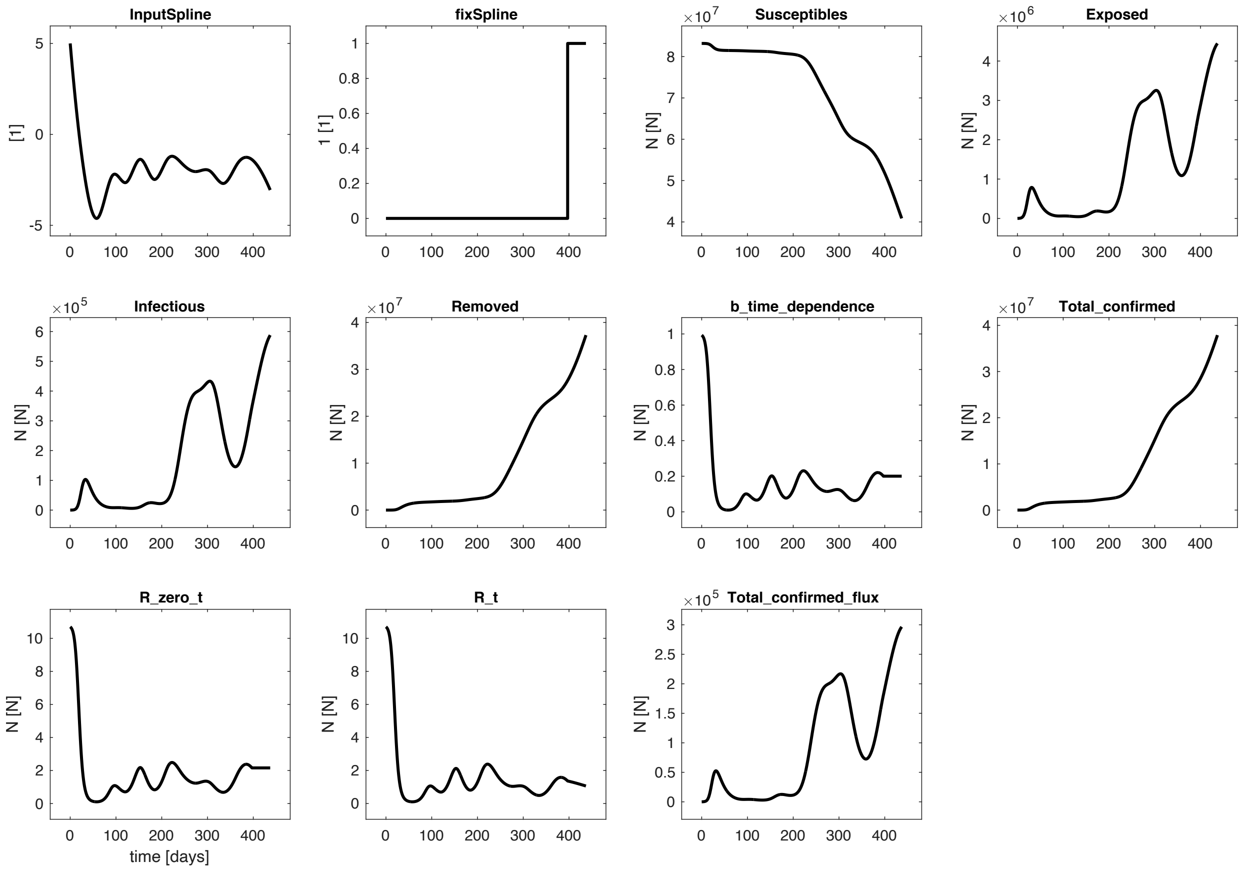

Figure 2: **Time courses of states and derived quantities**
All states and derived variables from the above model definition are plotted here. Over time, the `Susceptibles` get move though `Exposed` and `Infectious` states to reach the `Removed` state. The `InputSpline` is transformed to a factor function (`b_time_dependence`) and is fixed at time of the last data point by a Heaviside function (1-`fixSpline`), as can be seen by the first two panels. The difference between `R_zero_t` and `R_t` is whether or not they factor in the depletion of `Susceptibles` or not. The `Total_confirmed_flux` variable it the one eventually entering the observation function and is therefore used for calibrating the system.

5.2 Fit to data

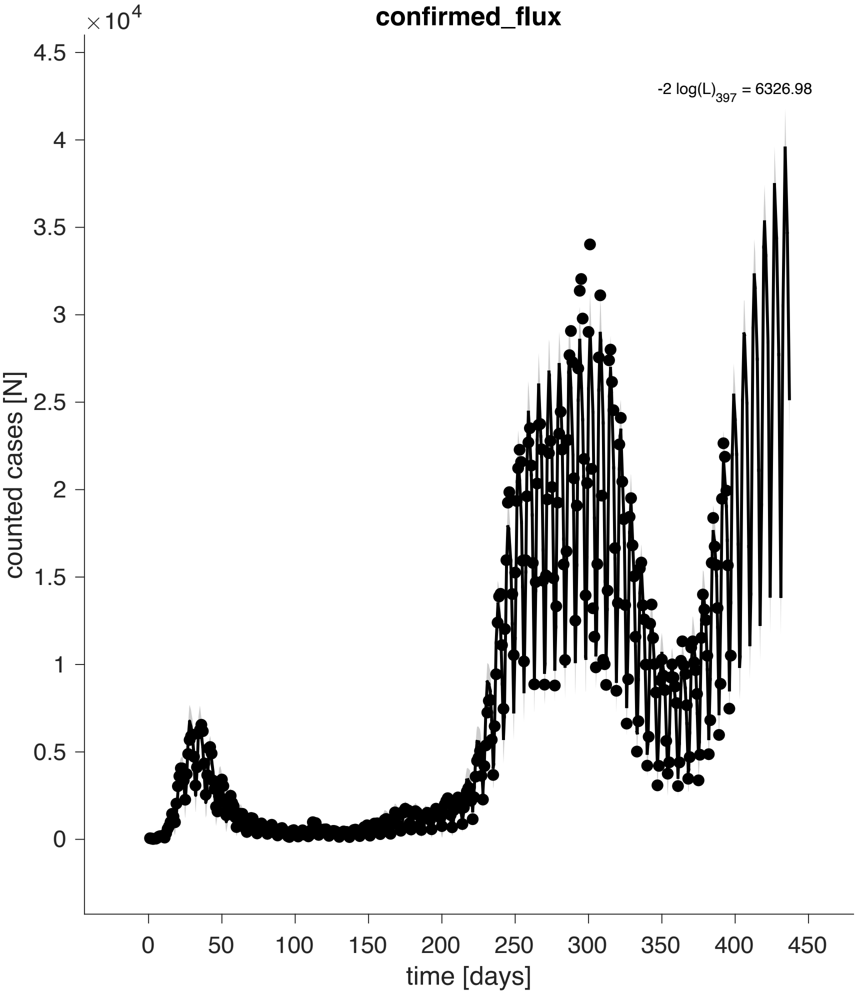

Figure 3: **Fitted incidence data**
The daily data (black dots) from Robert-Koch-Institut about reported positive cases of Covid-19 is fitted (black line) by the observation function of the dynamic model given in section 4.7. Time point zero is the first day where a total of 100 confirmed cases has been reached. The displayed shadow visualizes the measurement noise that is estimated based on Poisson error model.

5.3 Parameter estimation and confidence intervals

In total 19 parameters are estimated from the experimental data. The best fit yields a value of the objective function $-2\log(L) = 6326.98$ for a total of 397 data points. The model parameters were estimated by maximum likelihood estimation. In Table 3 the estimated parameter values are given. In Table 4, 95% confidence intervals for the estimated parameter values derived by the profile likelihood method as shown in figure 4 are given.

	name	θ_{min}	$\hat{\theta}$	θ_{max}	log	non-log $\hat{\theta}$	estimated
1	AA_0	-3	+0.0000	+5	1	$+1.00 \cdot 10^{+00}$	0
2	AA_1	-5	-0.0775	+3	1	$+8.37 \cdot 10^{-01}$	1
3	AA_2	-5	-0.6235	+3	1	$+2.38 \cdot 10^{-01}$	1
4	AA_3	-5	-1.2943	+3	1	$+5.08 \cdot 10^{-02}$	1
5	R_null	+0.1	+1.0321	+1	1	$+1.08 \cdot 10^{+01}$	1
6	init_Infectious_Germany	+0	+1.9500	+4	1	$+8.91 \cdot 10^{+01}$	1
7	phi_1	-6	+0.4263	+1e+01	0	$+4.26 \cdot 10^{-01}$	1
8	phi_2	-6	+4.5821	+1e+01	0	$+4.58 \cdot 10^{+00}$	1
9	phi_3	-6	+1.3386	+1e+01	0	$+1.34 \cdot 10^{+00}$	1
10	rate_infectious	-1	-1.1761	+0	1	$+6.67 \cdot 10^{-02}$	1
11	rate_removal	-1	-0.3010	-0.3	1	$+5.00 \cdot 10^{-01}$	1
12	scaleCon	-1	-1.0000	+0	1	$+1.00 \cdot 10^{-01}$	1
13	sigma	-1	+1.1220	+1	1	$+1.32 \cdot 10^{+01}$	1
14	spValue10	-5	-2.0460	+5	0	$-2.05 \cdot 10^{+00}$	1
15	spValue11	-5	-2.0144	+5	0	$-2.01 \cdot 10^{+00}$	1
16	spValue12	-5	-2.6938	+5	0	$-2.69 \cdot 10^{+00}$	1
17	spValue13	-5	-1.5725	+5	0	$-1.57 \cdot 10^{+00}$	1
18	spValue14	-5	-3.6622	+5	0	$-3.66 \cdot 10^{+00}$	1
19	spValue2	-5	-2.1203	+5	0	$-2.12 \cdot 10^{+00}$	0
20	spValue3	-5	-4.5711	+5	0	$-4.57 \cdot 10^{+00}$	0
21	spValue4	-5	-2.2842	+5	0	$-2.28 \cdot 10^{+00}$	0
22	spValue5	-5	-2.6422	+5	0	$-2.64 \cdot 10^{+00}$	0
23	spValue6	-5	-1.3728	+5	0	$-1.37 \cdot 10^{+00}$	0
24	spValue7	-5	-2.4758	+5	0	$-2.48 \cdot 10^{+00}$	0
25	spValue8	-5	-1.3232	+5	0	$-1.32 \cdot 10^{+00}$	1
26	spValue9	-5	-1.6074	+5	0	$-1.61 \cdot 10^{+00}$	1
27	tLast	+1	+397.0000	+4e+02	0	$+3.97 \cdot 10^{+02}$	0

Table 3: **Estimated parameter values**
$\hat{\theta}$ indicates the estimated value of the parameters. θ_{min} and θ_{max} indicate the upper and lower bounds for the parameters. The log-column indicates if the value of a parameter was log-transformed. If log $\equiv 1$ the non-log-column indicates the non-logarithmic value of the estimate. The estimated-column indicates if the parameter value was estimated (1), or was fixed (0).

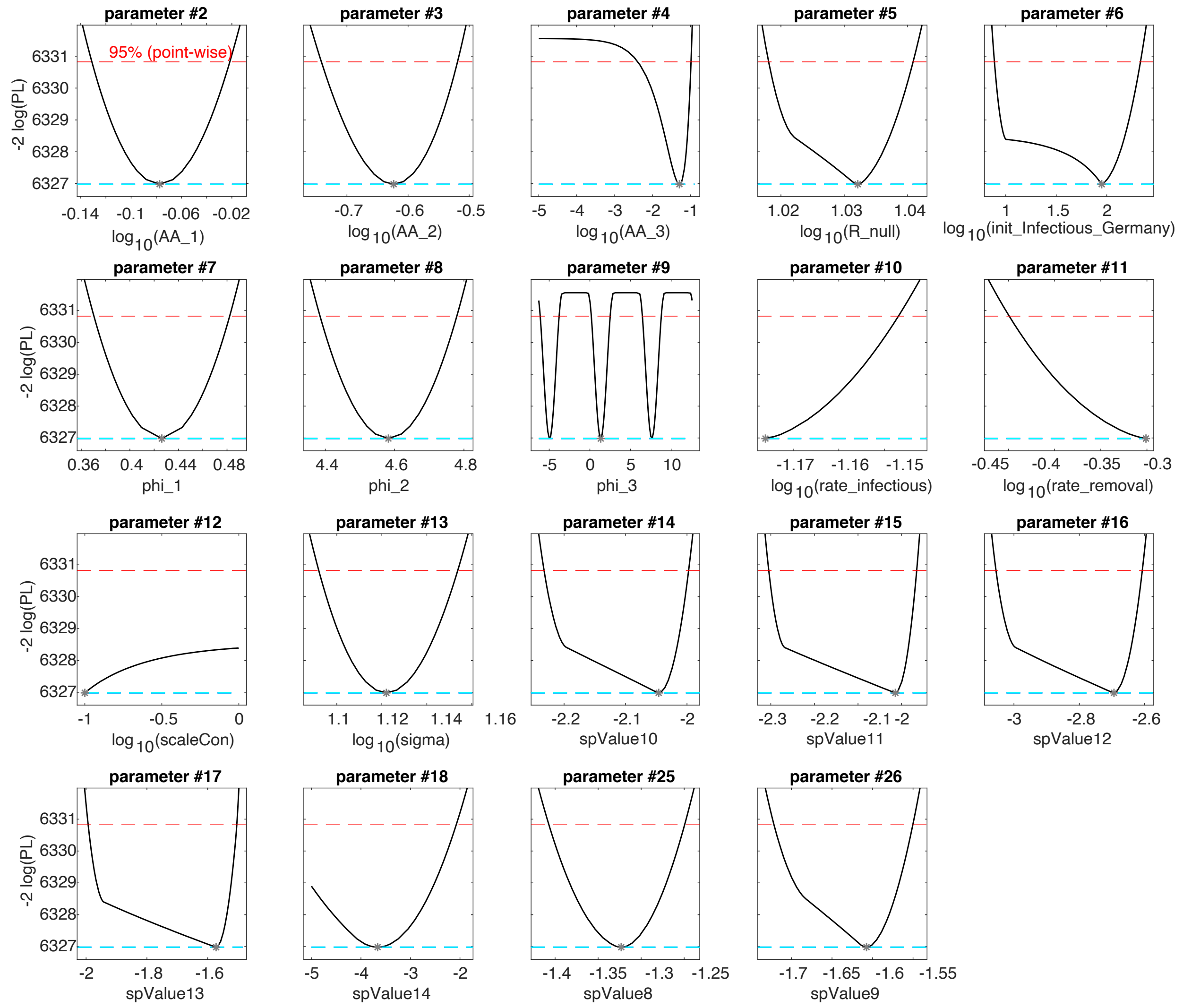

Figure 4: **Parameter profiles**
Uncertainty in predictions is determined by uncertainty in estimated parameters which can be calculated by profile likelihood analysis. Parameters `AA_N` and `phi_N` (`N` = 1,2,3) determine the modeulation factor per weekday (see figure 21 of the supplement). Spline Parameters `spValueN` (`N` = 8,...,14) resemble those spline parameters that will affect uncertainty in predictions. The first spline parameters will only affect the fit of the early time course, and not the predictions. Confidence intervals are determined by the profile intersecting the threshold, see table 4.

	name	$\hat{\theta}$	σ^-	σ^+
2	AA_1	-0.077	-0.131	-0.022
3	AA_2	-0.624	-0.742	-0.519
4	AA_3	-1.294	-2.396	-0.989
5	R_null	+1.032	+1.018	+1.041
6	init_Infectious_Germany	+1.950	+0.888	+2.334
7	phi_1	+0.426	+0.370	+0.482
8	phi_2	+4.582	+4.385	+4.779
9	phi_3	+1.339	-6.106	+8.787
10	rate_infectious	-1.176	-Inf	-1.147
11	rate_removal	-0.301	-0.449	+Inf
12	scaleCon	-1.000	-Inf	+Inf
13	sigma	+1.122	+1.092	+1.154
14	spValue10	-2.046	-2.233	-1.997
15	spValue11	-2.014	-2.305	-1.965
16	spValue12	-2.694	-3.055	-2.607
17	spValue13	-1.572	-1.993	-1.504
18	spValue14	-3.662	-Inf	-2.075
25	spValue8	-1.323	-1.407	-1.249
26	spValue9	-1.607	-1.722	-1.550

Table 4: **Confidence intervals for the estimated parameter values derived by the profile likelihood**

$\hat{\theta}$ indicates the estimated optimal parameter value. σ^- and σ^+ indicate 95% point-wise confidence intervals. A value of ±Inf are displayed if the bound of parameter estimation is hit by the profile likelihood algorithm. See figure 4 of the supplement for the corresponding profiles.

6 Results for federal states

6.1 Predictions for all federal states

Analogous to figure 2 of the main paper, we present here the independent results for all 16 federal states. Note the different scales of estimated effective $R(t)$ which might point to a degree of freedom that is not incorporated in the data. Further investigation about this model feature might be required. The caption of the figure is repeated here:

The incidence data of the entire time course is fitted (panel a) to estimate all dynamic parameters including the time-dependent infection rate that corresponds to $R(t)$(panel d). Predictions of incidences (panels b and c) and derived quantities (panels e and f) for a zoomed in time span are shown. 95%-confidence intervals (color-shaded areas) are inferred by profile likelihood calculation.

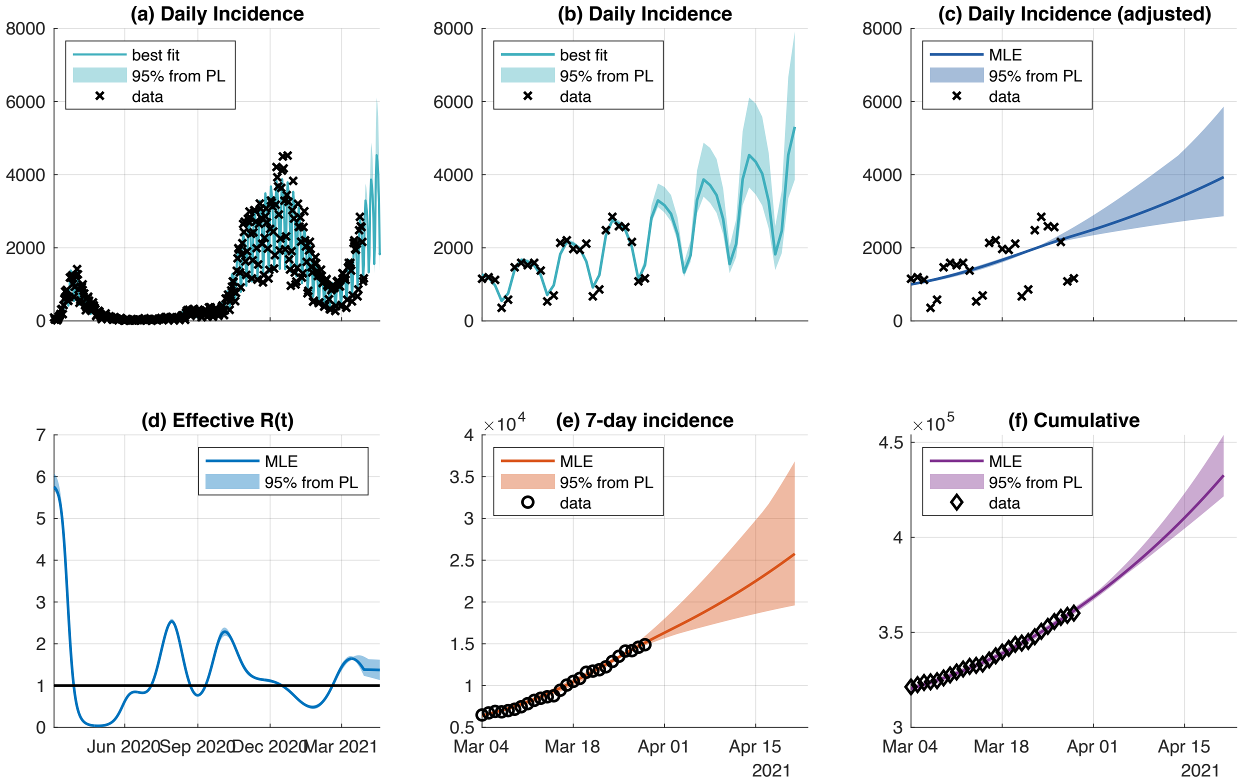

Figure 5: **Result for Baden-Wuerttemberg**
Corresponding result to figure 2 of the main paper. See page 14 for detailed description.

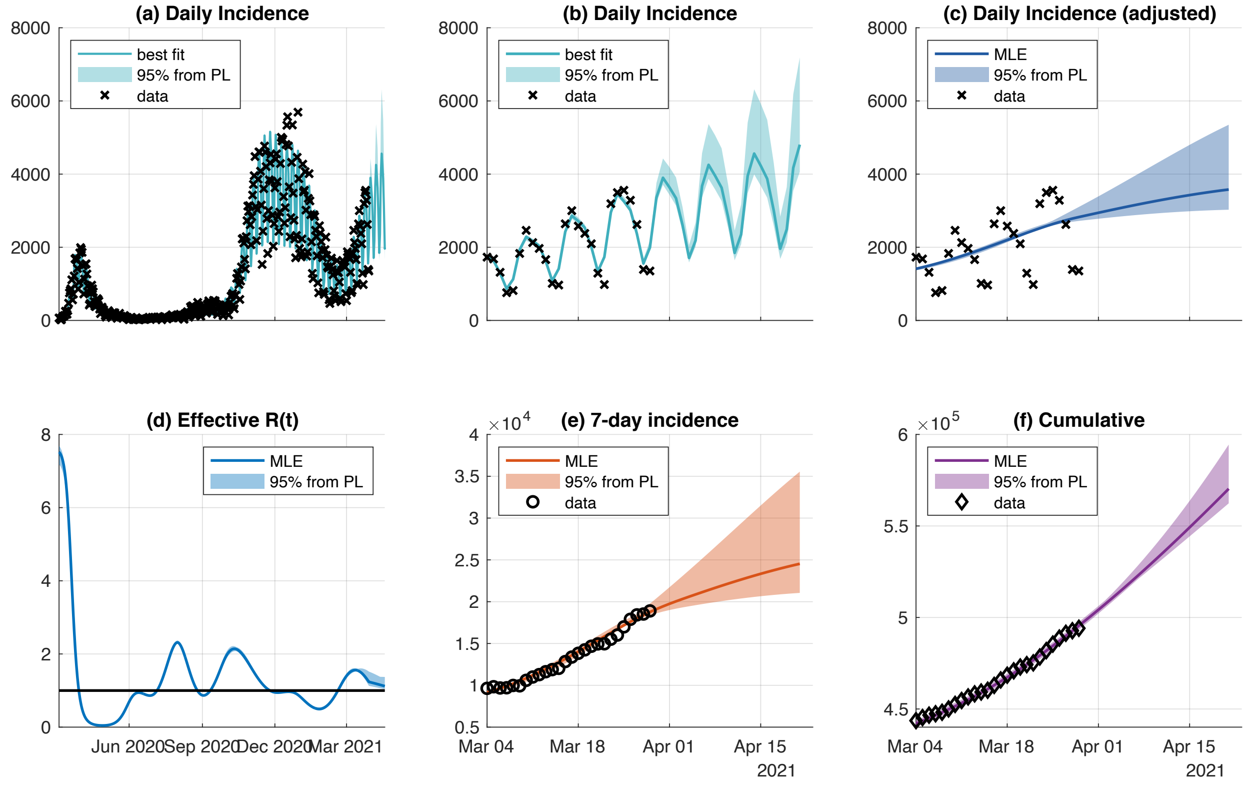

Figure 6: **Result for Bayern**
Corresponding result to figure 2 of the main paper. See page 14 for detailed description.

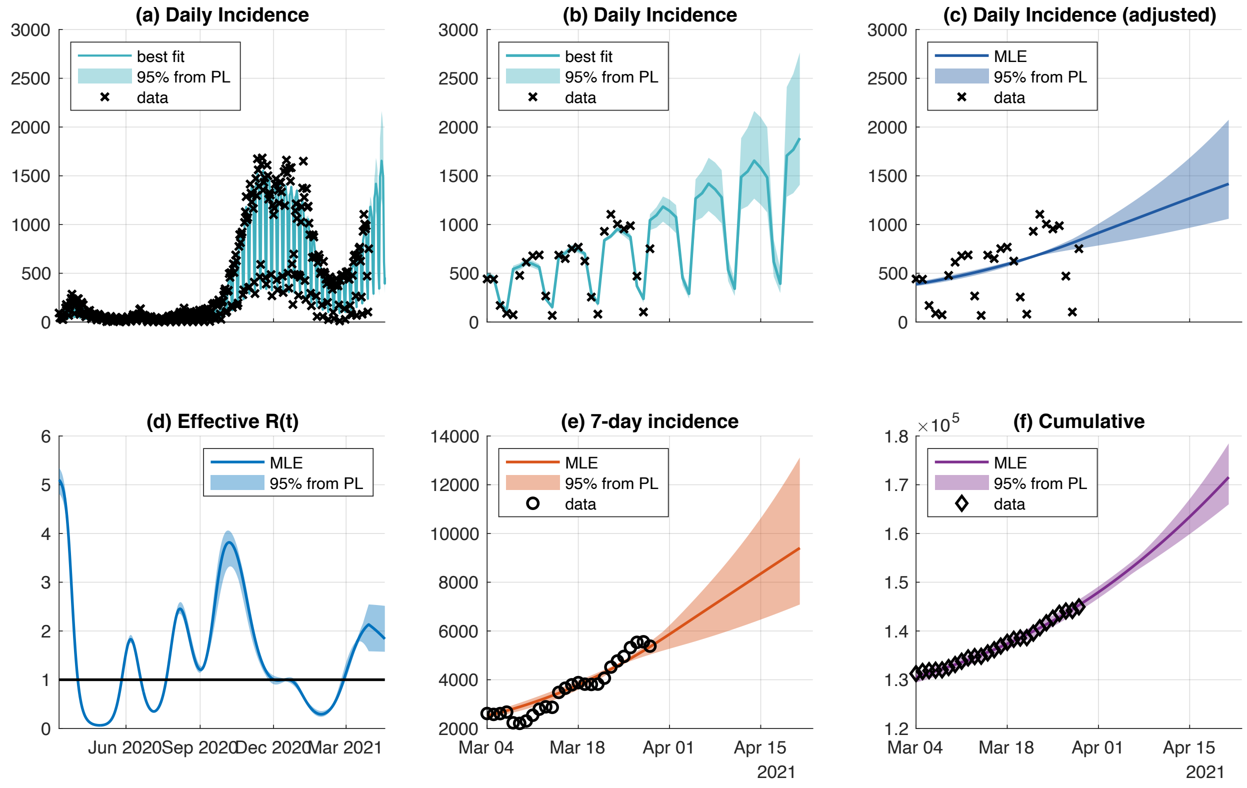

Figure 7: **Result for Berlin**
Corresponding result to figure 2 of the main paper. See page 14 for detailed description.

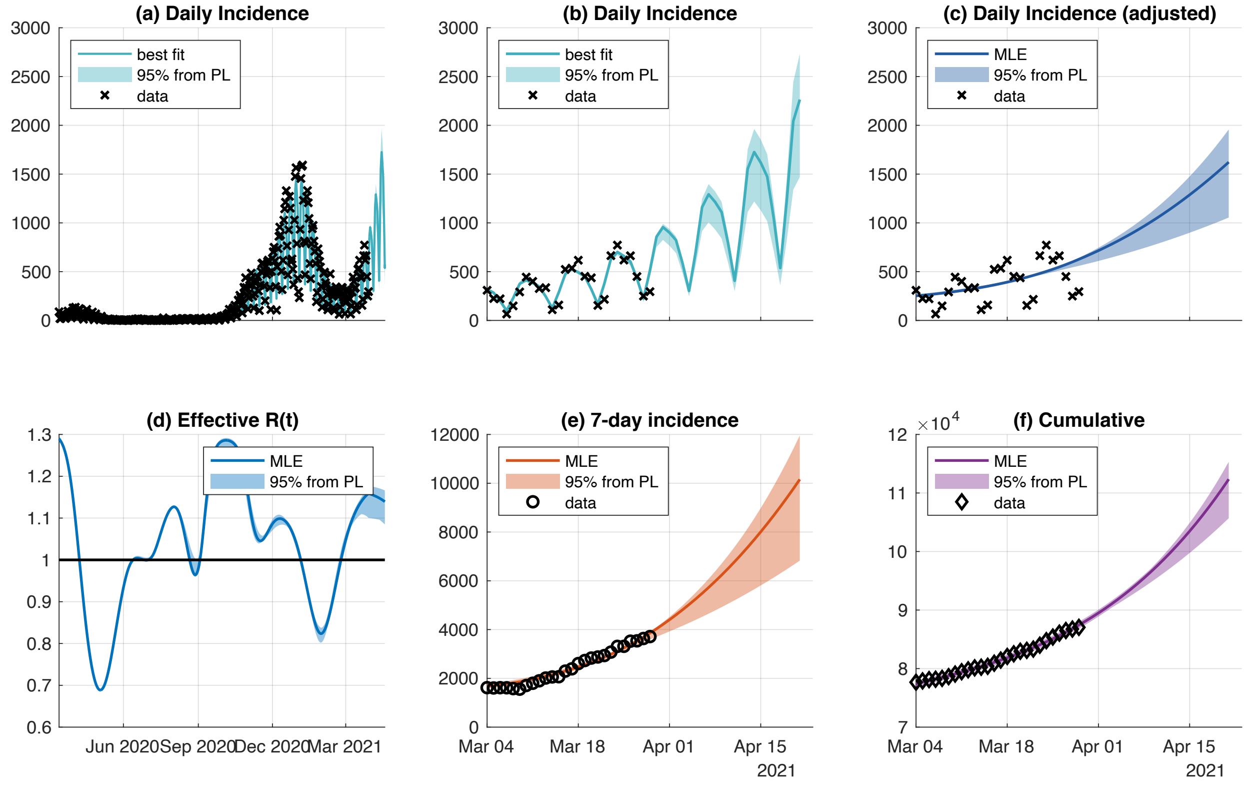

Figure 8: **Result for Brandenburg**
Corresponding result to figure 2 of the main paper. See page 14 for detailed description.

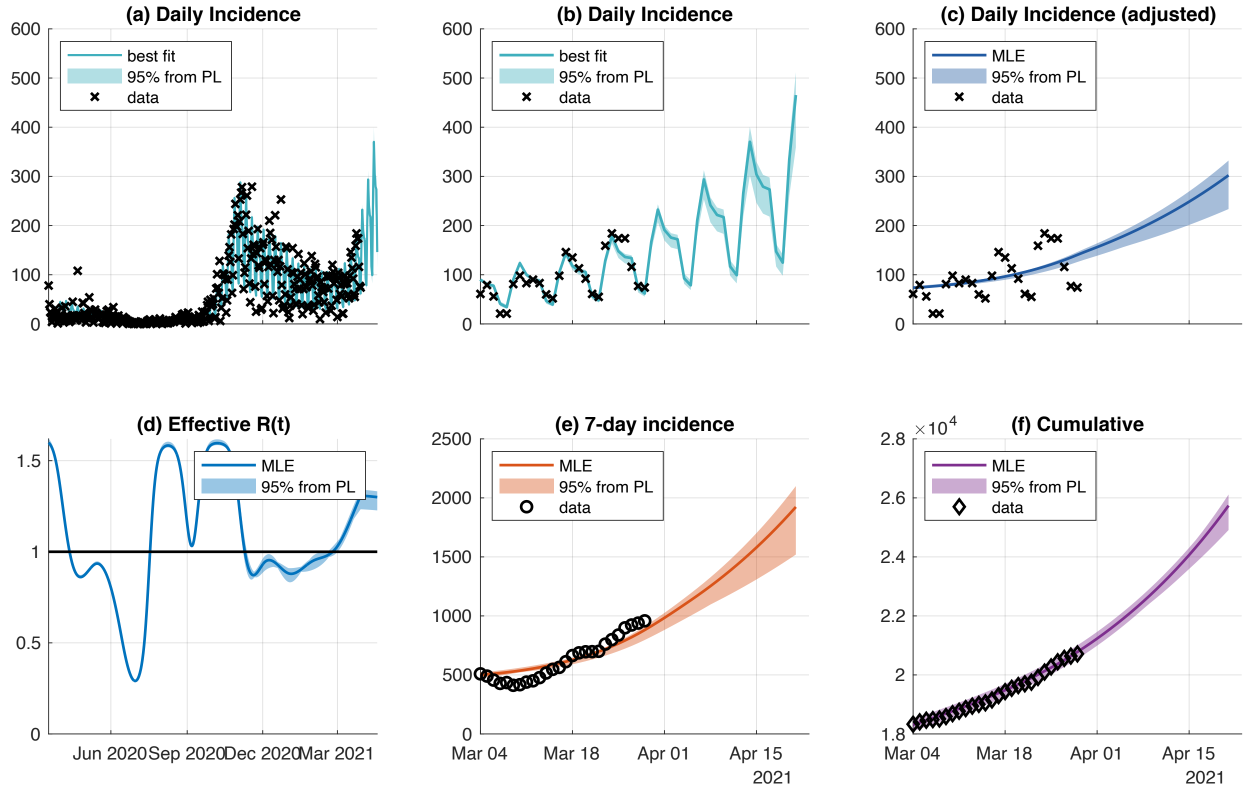

Figure 9: **Result for Bremen**
Corresponding result to figure 2 of the main paper. See page 14 for detailed description.

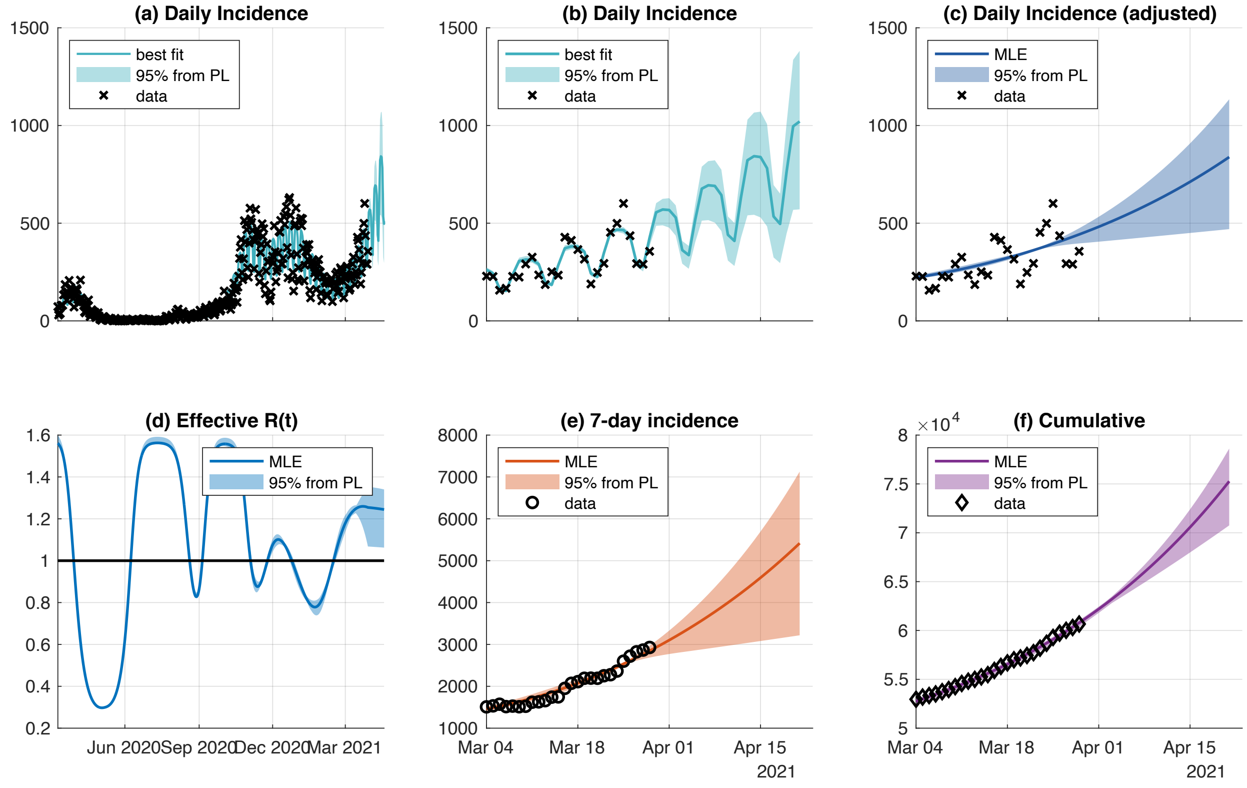

Figure 10: **Result for Hamburg**
Corresponding result to figure 2 of the main paper. See page 14 for detailed description.

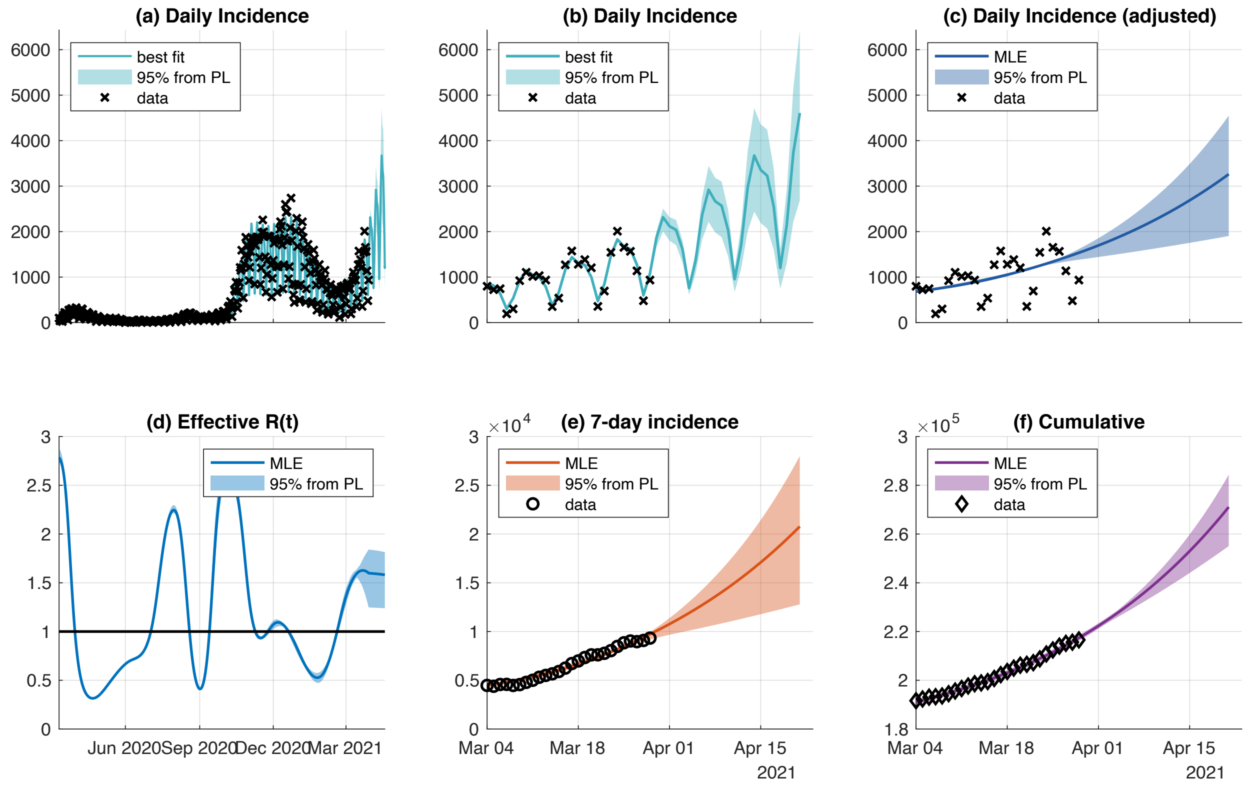

Figure 11: **Result for Hessen**
Corresponding result to figure 2 of the main paper. See page 14 for detailed description.

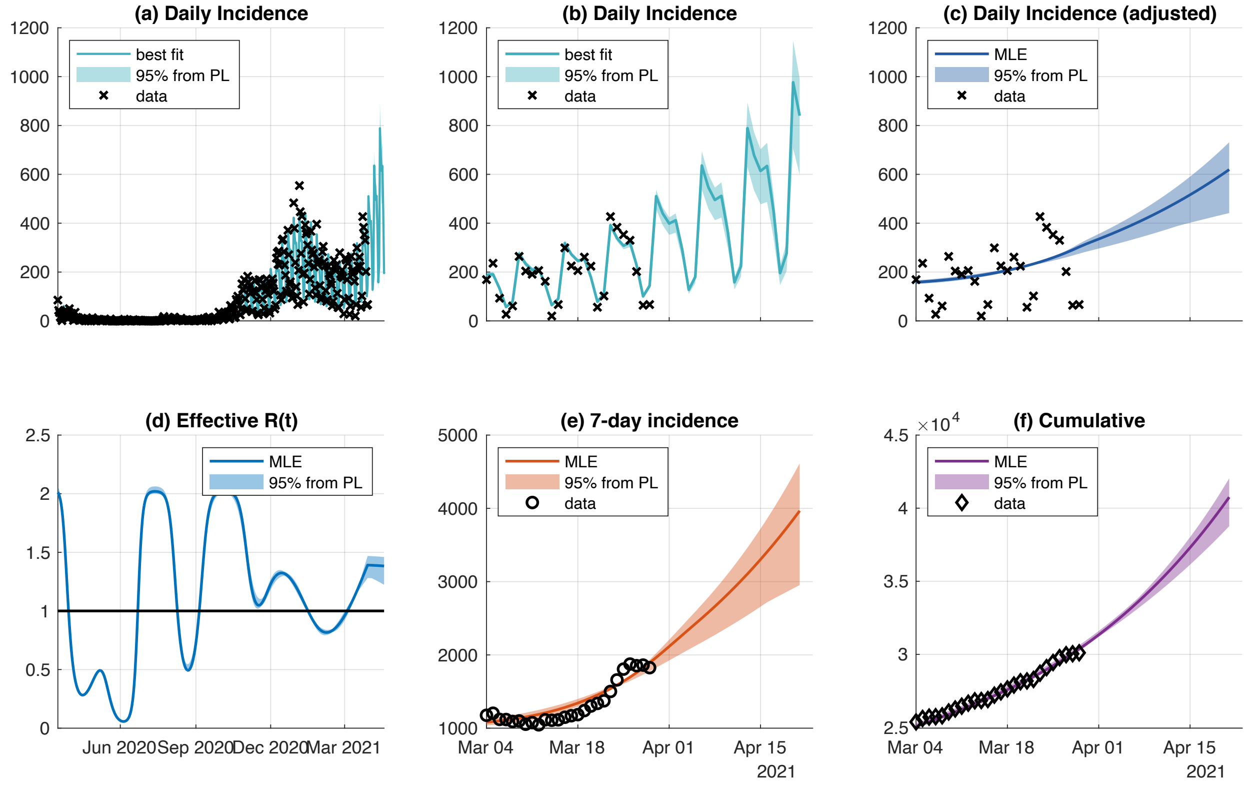

Figure 12: **Result for Mecklenburg-Vorpommern**
Corresponding result to figure 2 of the main paper. See page 14 for detailed description.

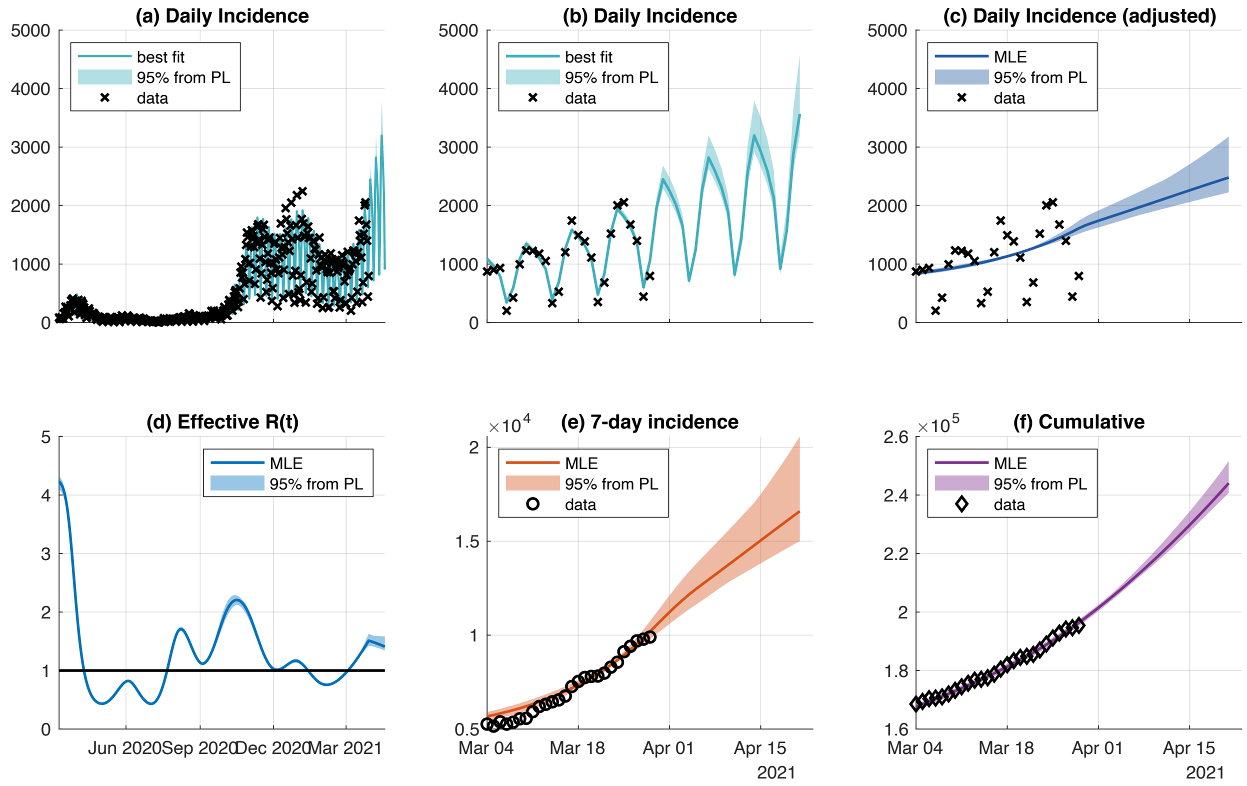

Figure 13: **Result for Niedersachsen**
Corresponding result to figure 2 of the main paper. See page 14 for detailed description.

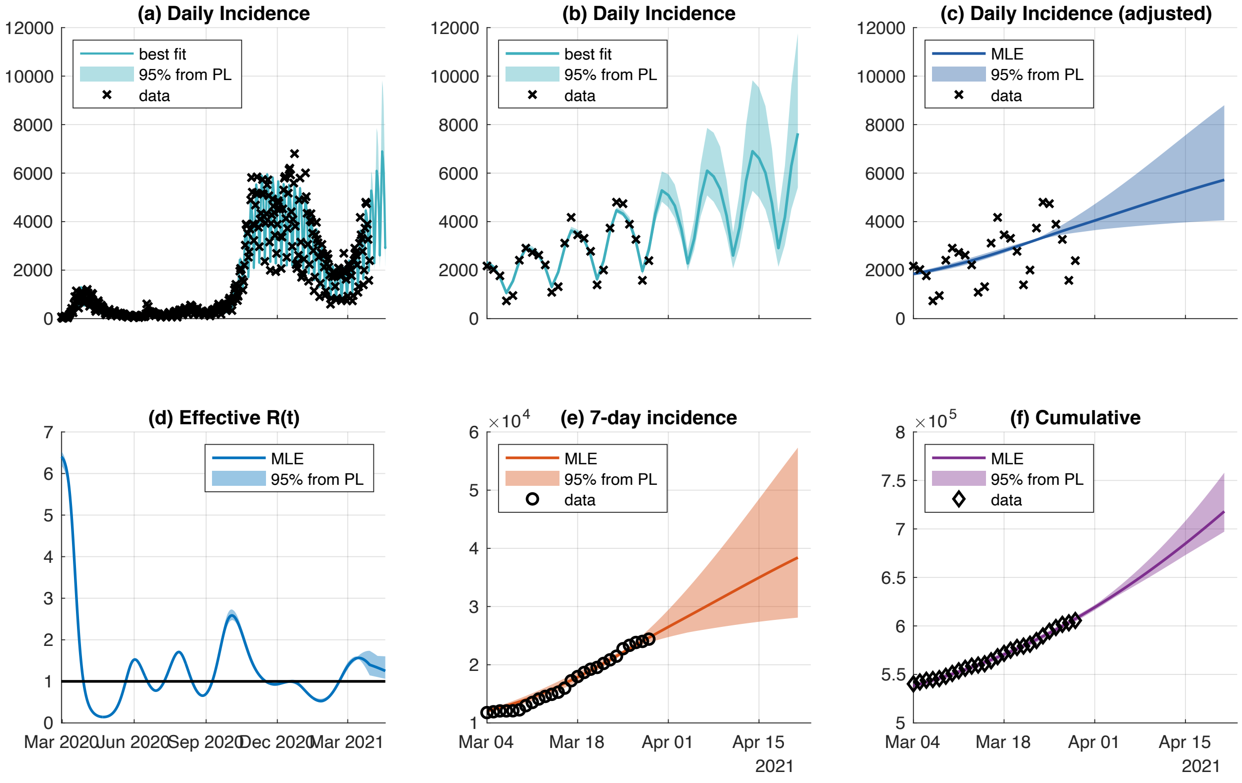

Figure 14: **Result for Nordrhein-Westfalen**
Corresponding result to figure 2 of the main paper. See page 14 for detailed description.

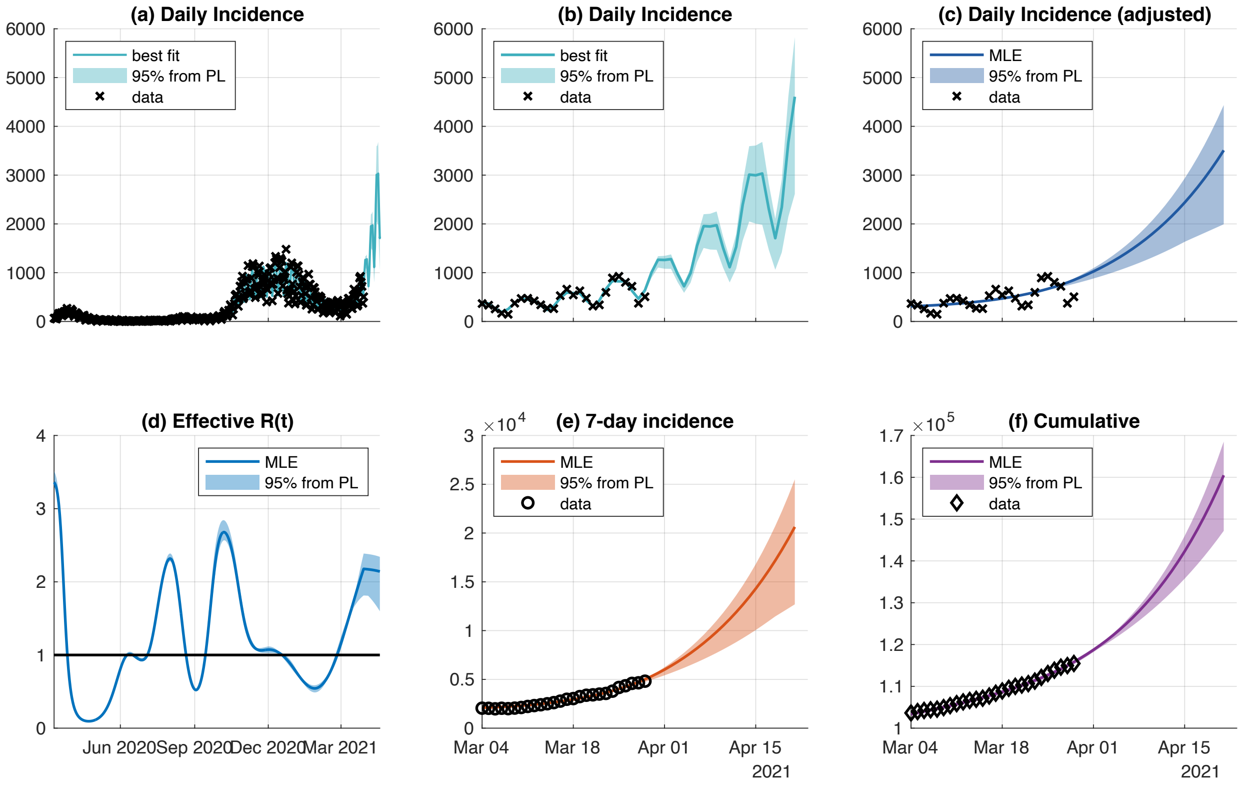

Figure 15: **Result for Rheinland-Pfalz**
Corresponding result to figure 2 of the main paper. See page 14 for detailed description.

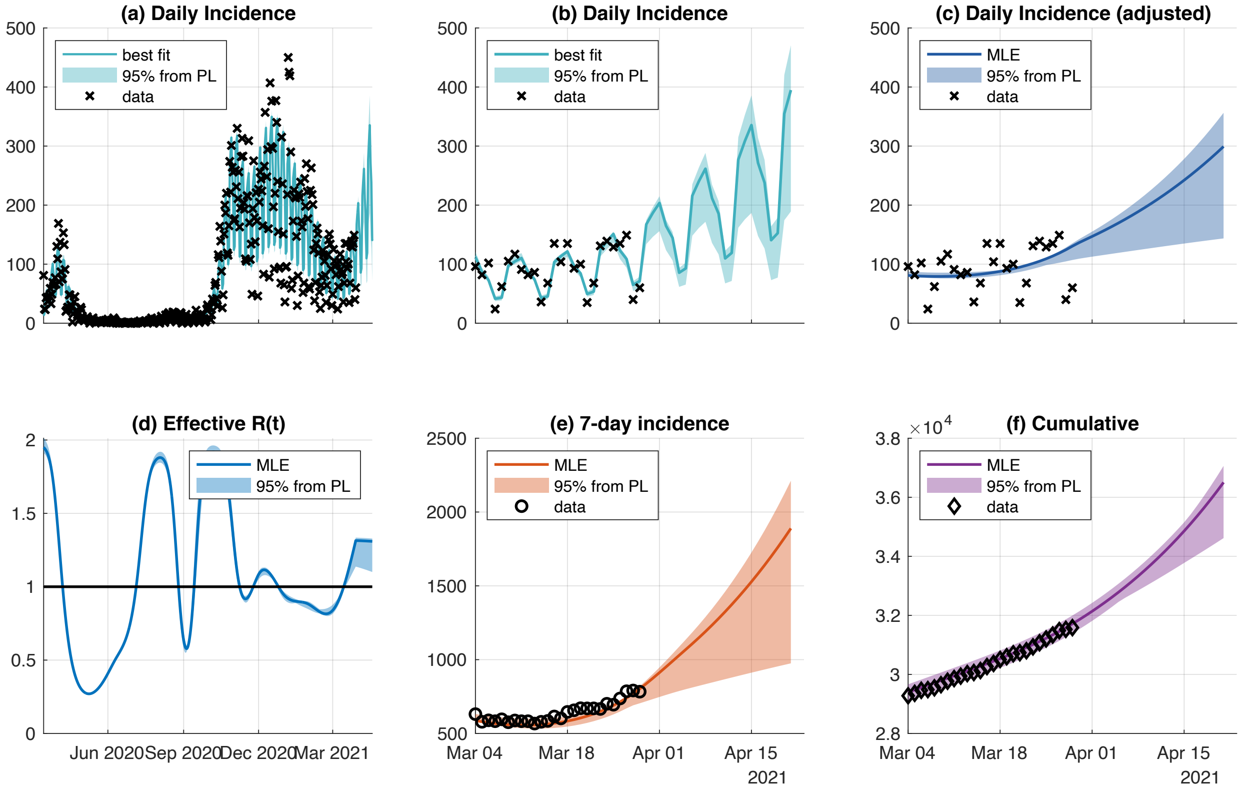

Figure 16: **Result for Saarland**
Corresponding result to figure 2 of the main paper. See page 14 for detailed description.

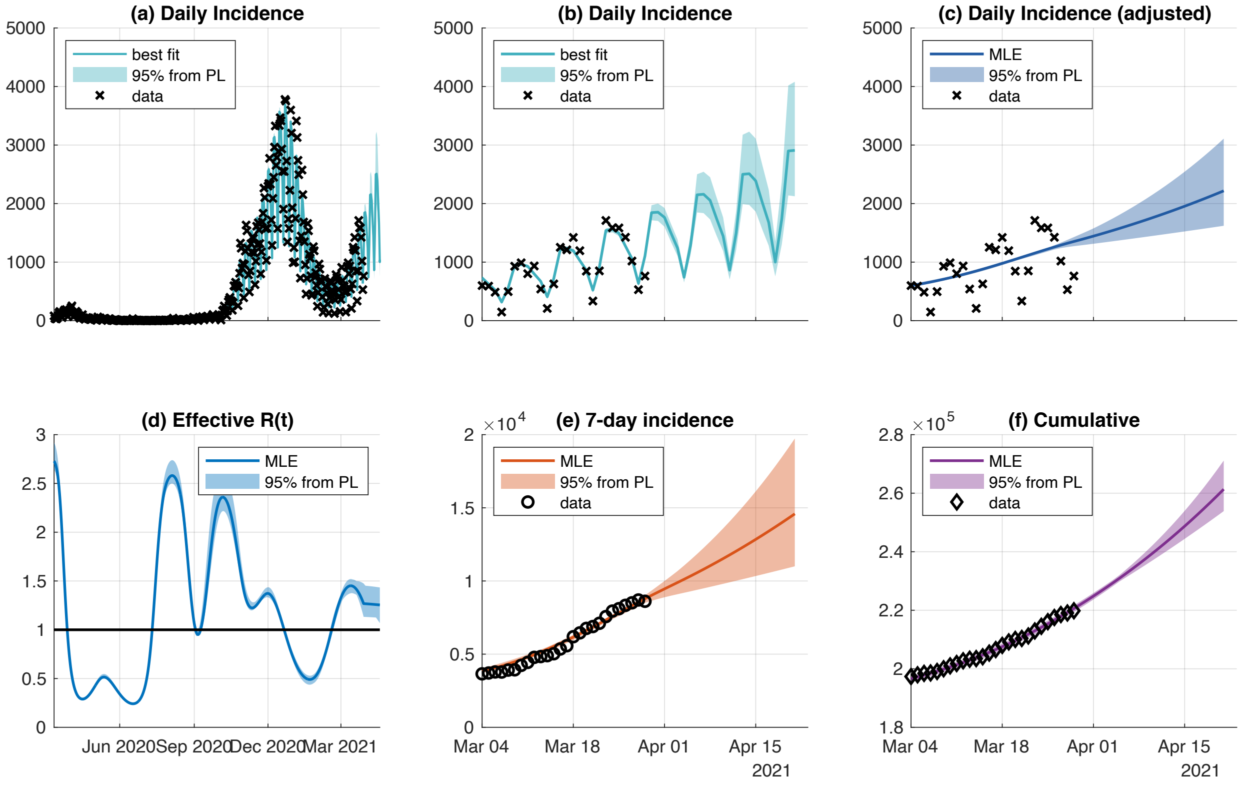

Figure 17: **Result for Sachsen**
Corresponding result to figure 2 of the main paper. See page 14 for detailed description.

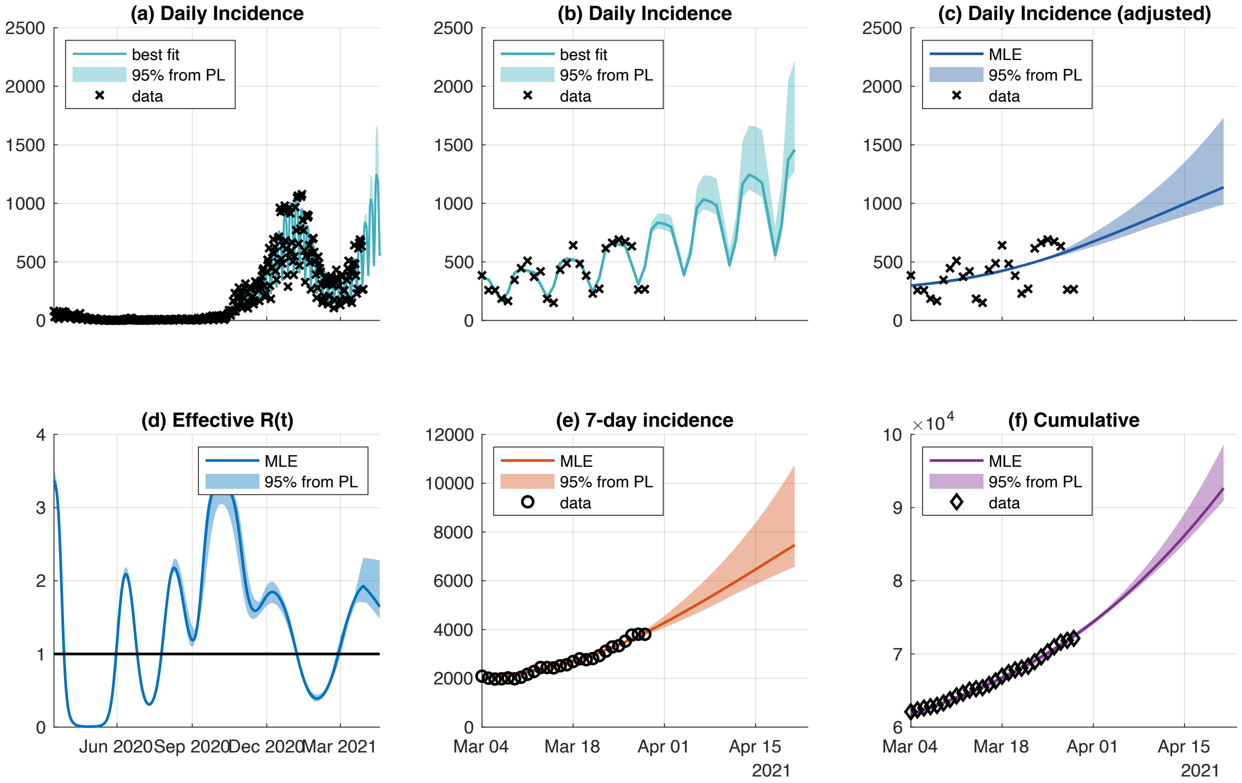

Figure 18: **Result for Sachsen-Anhalt**
Corresponding result to figure 2 of the main paper. See page 14 for detailed description.

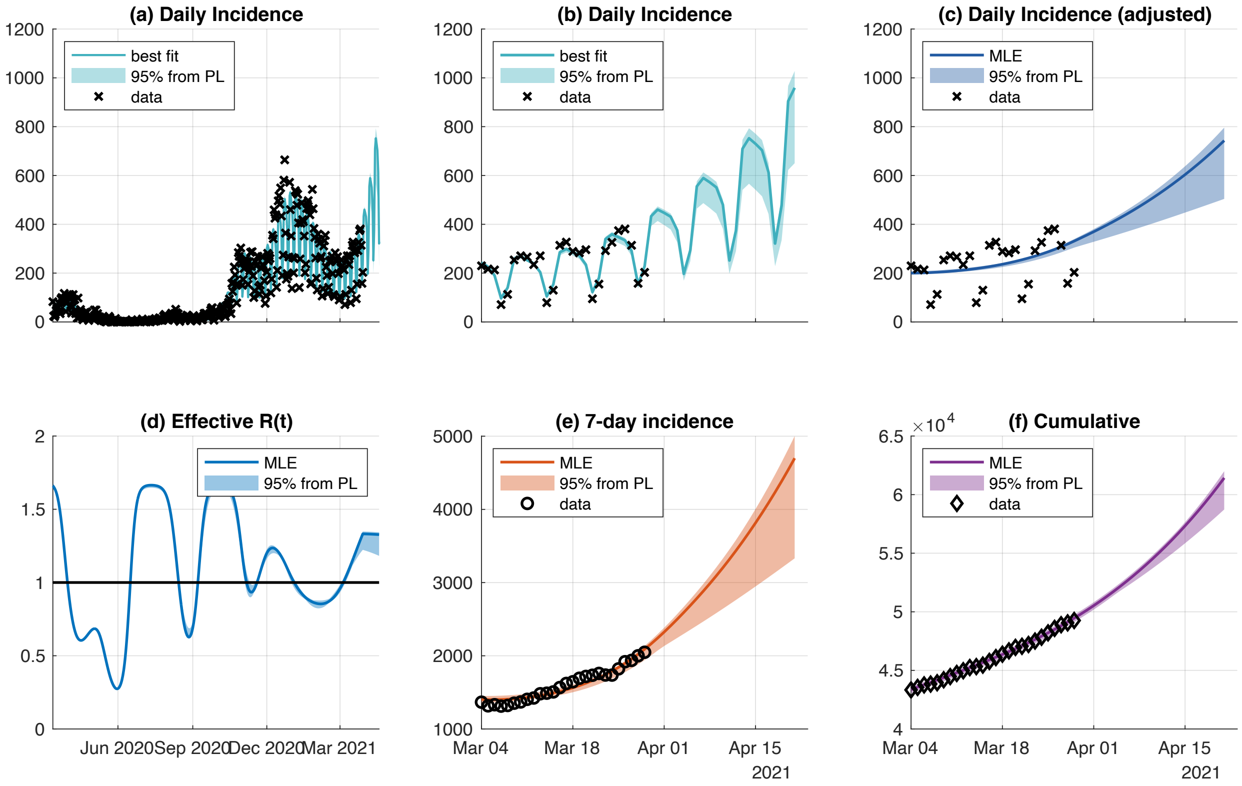

Figure 19: **Result for Schleswig-Holstein**
Corresponding result to figure 2 of the main paper. See page 14 for detailed description.

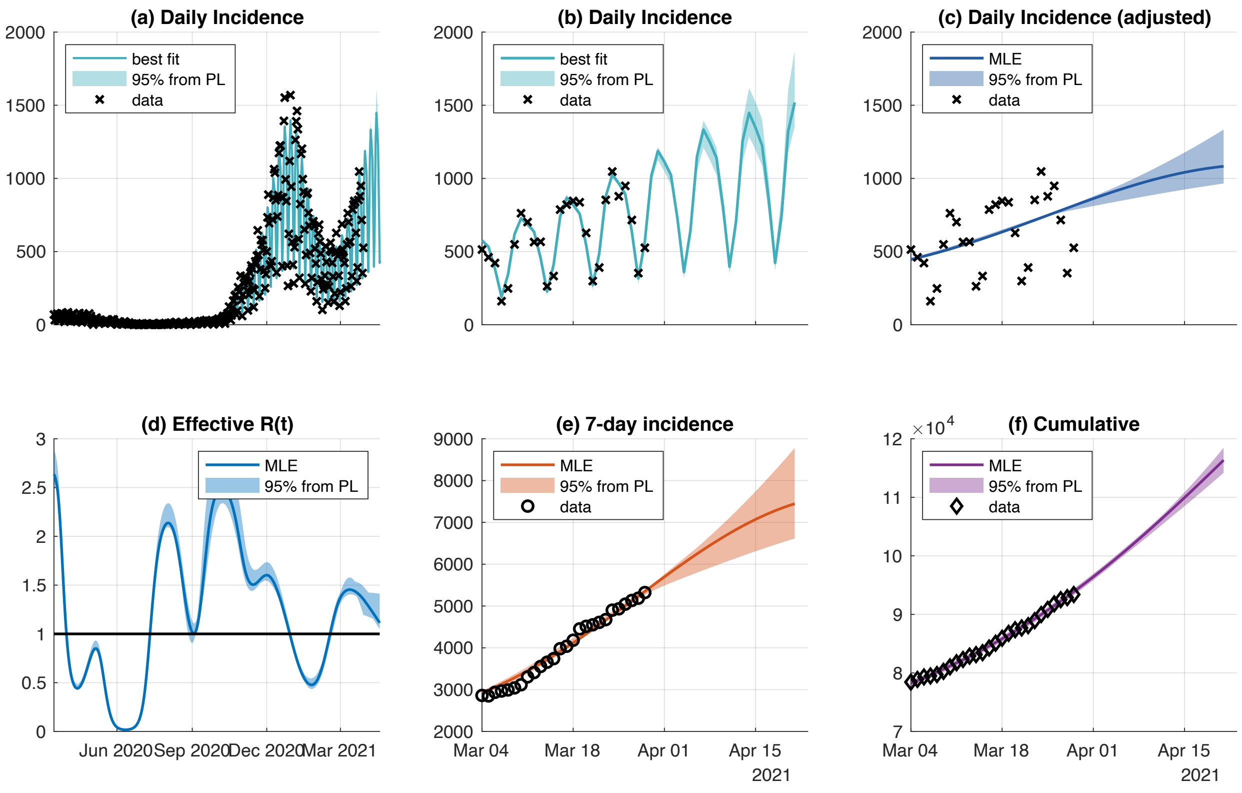

Figure 20: **Result for Thueringen**
Corresponding result to figure 2 of the main paper. See page 14 for detailed description.

6.2 Weekly factor

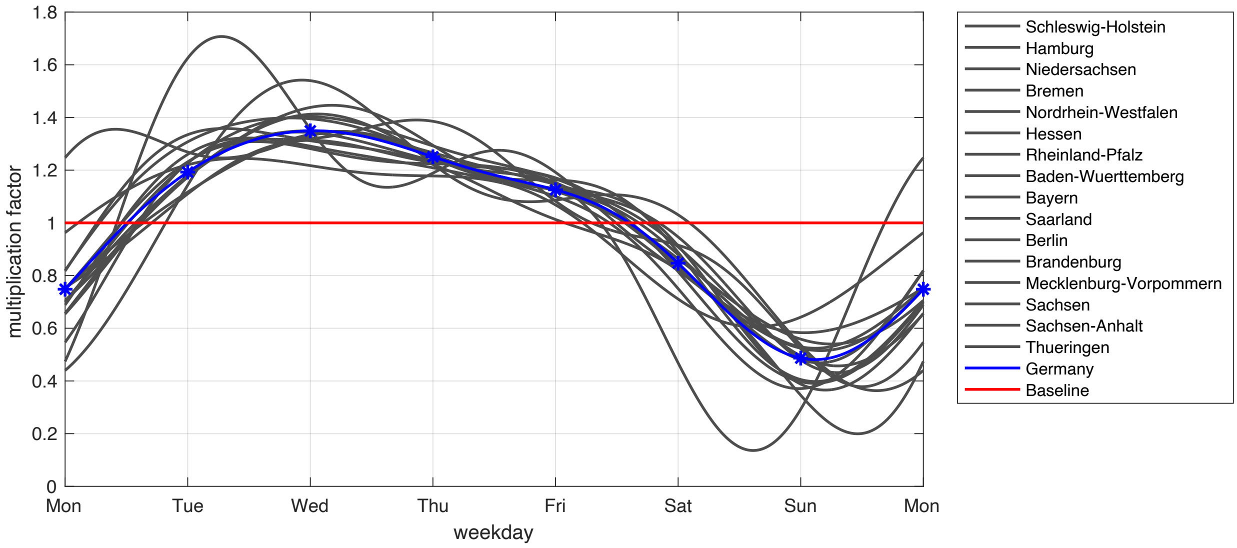

Figure 21: **Modulation factor is dependent on federal state**
Due to the parametrisation via a Fourier series, the weekday modulation function is a periodic function with periodicity 7. Estimates for all federal states (grey lines) and Germany (blue line) are computed independent of each other. Albeit continuous function are estimated, only the function evaluation at seven discrete time points enter the observation function (blue asterisks). The baseline value of 1 (red line) indicates no effect by the weekday. Also, compare figure 1 of this Supplement where the respective data features of Germany are visualised.

7 Results for all regions and counties: Landkreise

For all counties, the federal state's trajectory is scaled down and uncertainties for scaling down are computed. Due to computing power and time limitations as results should be computed with the latest data, this method was chosen and turned out to be feasible for daily computations of results. In the following figures we give an example of how results for the county level look like for the relevant time frame which is the most recent couple of months and the prediction for the upcoming weeks. For clarity, we display the trajectories without weekly modulation and after merging the two different approaches described in the main paper.

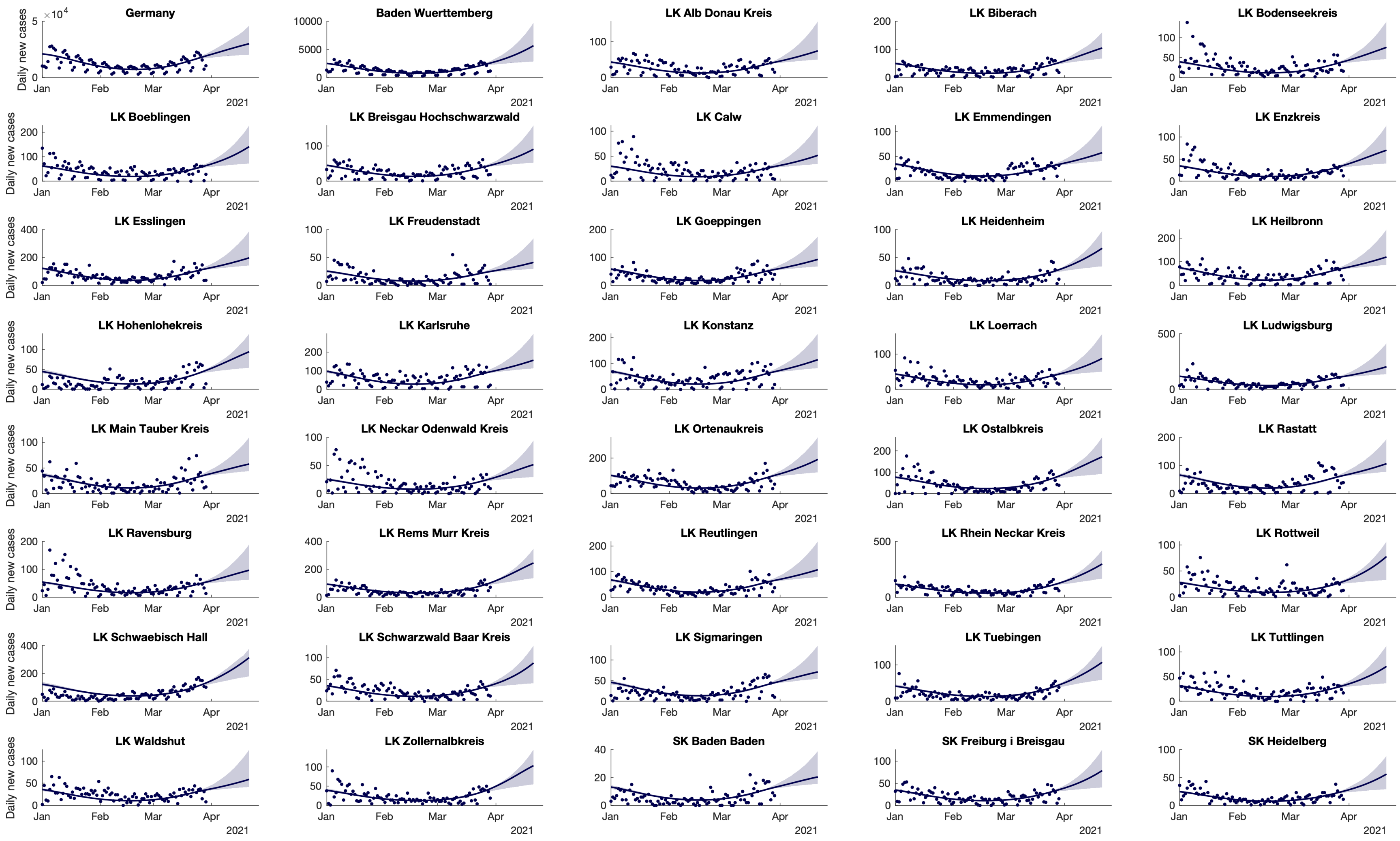
Germany
Baden Wuerttemberg
LK Alb Donau Kreis
LK Biberach
LK Bodenseekreis
LK Boeblingen
LK Breisgau Hochschwarzwald
LK Calw
LK Emmendingen
LK Enzkreis
LK Esslingen
LK Freudenstadt
LK Goeppingen
LK Heidenheim
LK Heilbronn
LK Hohenlohekreis
LK Karlsruhe
LK Konstanz
LK Loerrach
LK Ludwigsburg
LK Main Tauber Kreis
LK Neckar Odenwald Kreis
LK Ortenaukreis
LK Ostalbkreis
LK Rastatt
LK Ravensburg
LK Rems Murr Kreis
LK Reutlingen
LK Rhein Neckar Kreis
LK Rottweil
LK Schwaebisch Hall
LK Schwarzwald Baar Kreis
LK Sigmaringen
LK Tuebingen
LK Tuttlingen
LK Waldshut
LK Zollernalbkreis
SK Baden Baden
SK Freiburg i Breisgau
SK Heidelberg
Daily new cases
Jan
Feb
Mar
Apr
2021

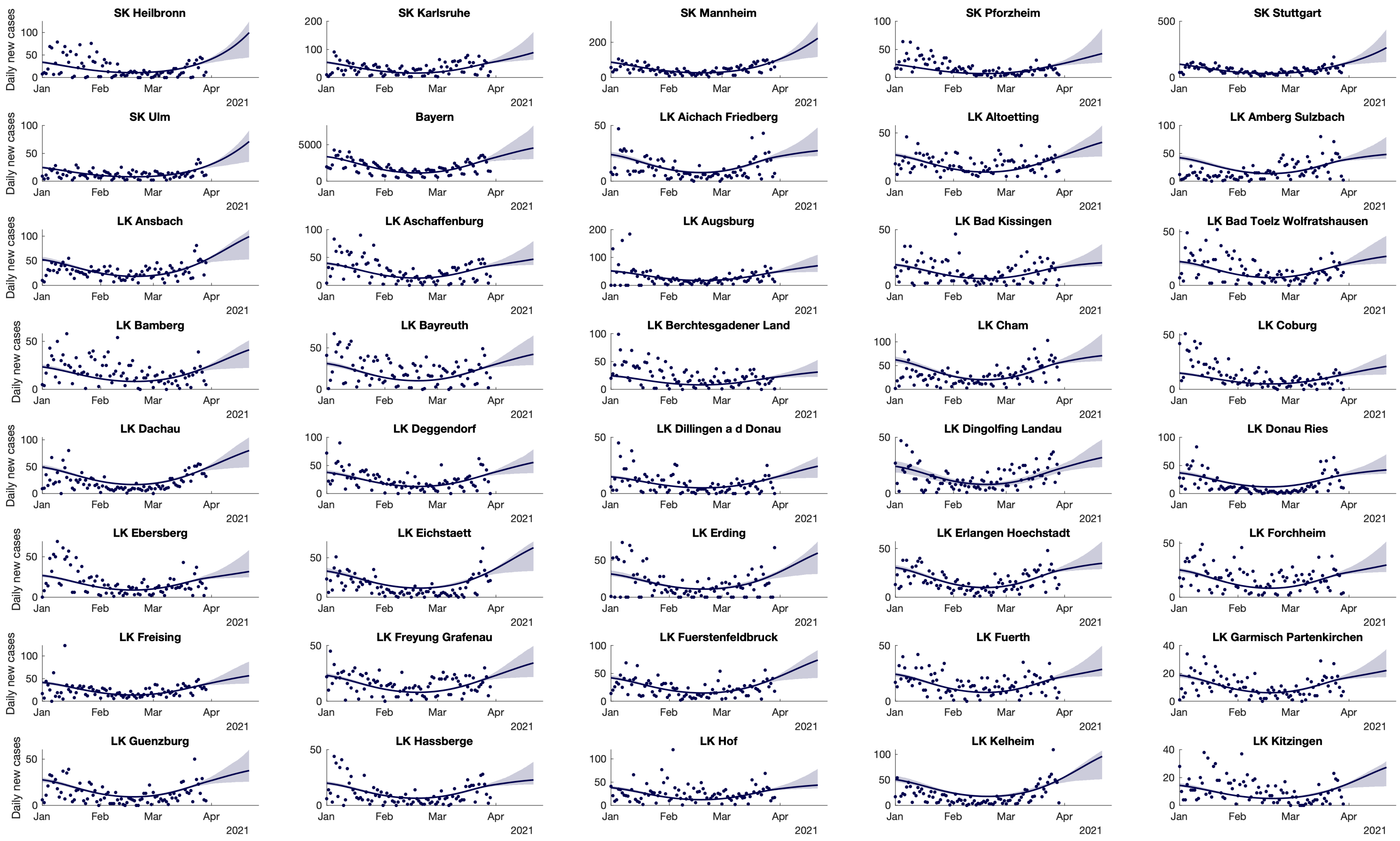
SK Heilbronn
SK Karlsruhe
SK Mannheim
SK Pforzheim
SK Stuttgart
SK Ulm
Bayern
LK Aichach Friedberg
LK Altoetting
LK Amberg Sulzbach
LK Ansbach
LK Aschaffenburg
LK Augsburg
LK Bad Kissingen
LK Bad Toelz Wolfratshausen
LK Bamberg
LK Bayreuth
LK Berchtesgadener Land
LK Cham
LK Coburg
LK Dachau
LK Deggendorf
LK Dillingen a d Donau
LK Dingolfing Landau
LK Donau Ries
LK Ebersberg
LK Eichstaett
LK Erding
LK Erlangen Hoechstadt
LK Forchheim
LK Freising
LK Freyung Grafenau
LK Fuerstenfeldbruck
LK Fuerth
LK Garmisch Partenkirchen
LK Guenzburg
LK Hassberge
LK Hof
LK Kelheim
LK Kitzingen
Daily new cases
Jan
Feb
Mar
Apr
2021

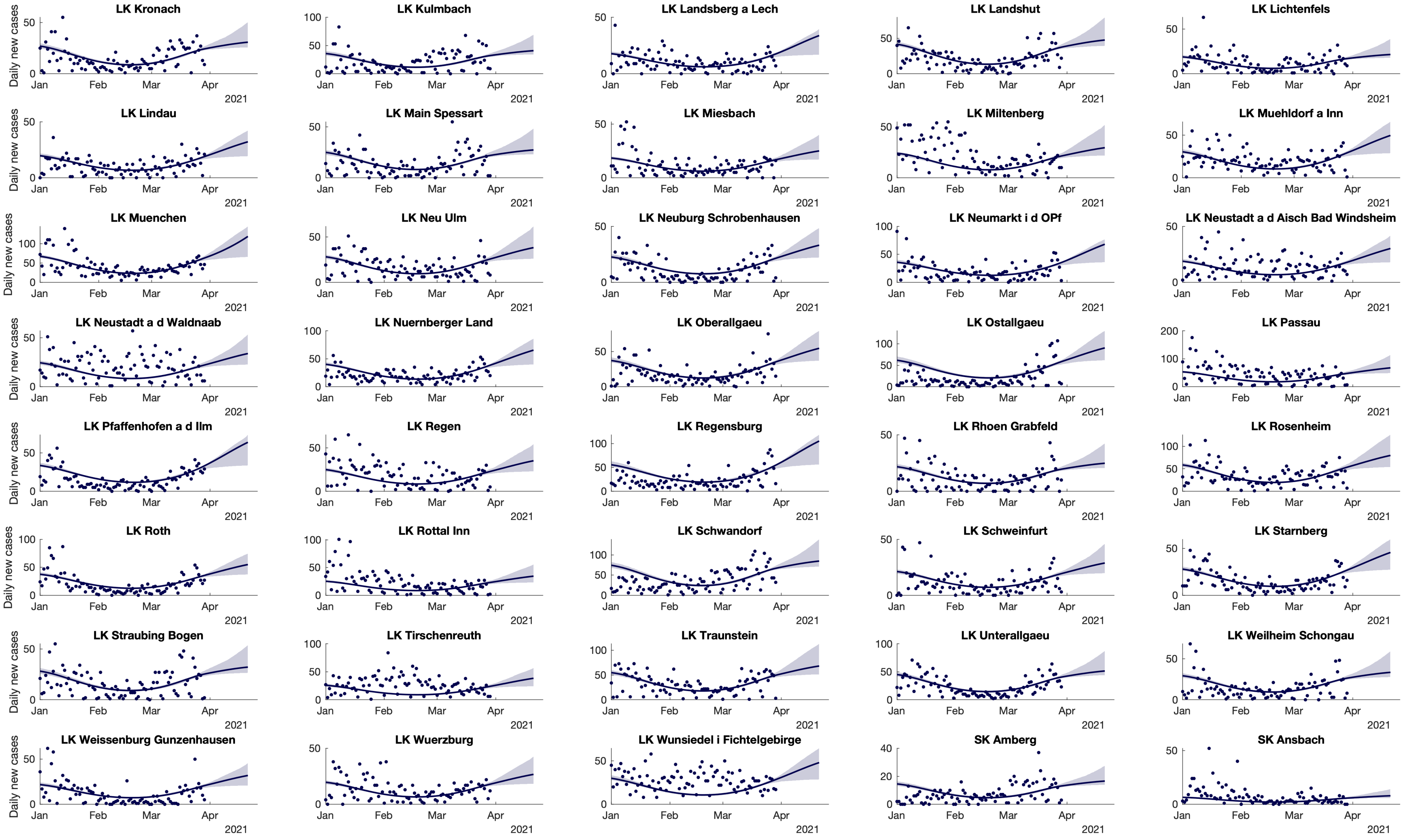
LK Kronach
LK Kulmbach
LK Landsberg a Lech
LK Landshut
LK Lichtenfels
LK Lindau
LK Main Spessart
LK Miesbach
LK Miltenberg
LK Muehldorf a Inn
LK Muenchen
LK Neu Ulm
LK Neuburg Schrobenhausen
LK Neumarkt i d OPf
LK Neustadt a d Aisch Bad Windsheim
LK Neustadt a d Waldnaab
LK Nuernberger Land
LK Oberallgaeu
LK Ostallgaeu
LK Passau
LK Pfaffenhofen a d Ilm
LK Regen
LK Regensburg
LK Rhoen Grabfeld
LK Rosenheim
LK Roth
LK Rottal Inn
LK Schwandorf
LK Schweinfurt
LK Starnberg
LK Straubing Bogen
LK Tirschenreuth
LK Traunstein
LK Unterallgaeu
LK Weilheim Schongau
LK Weissenburg Gunzenhausen
LK Wuerzburg
LK Wunsiedel i Fichtelgebirge
SK Amberg
SK Ansbach
Daily new cases
Jan
Feb
Mar
Apr
2021

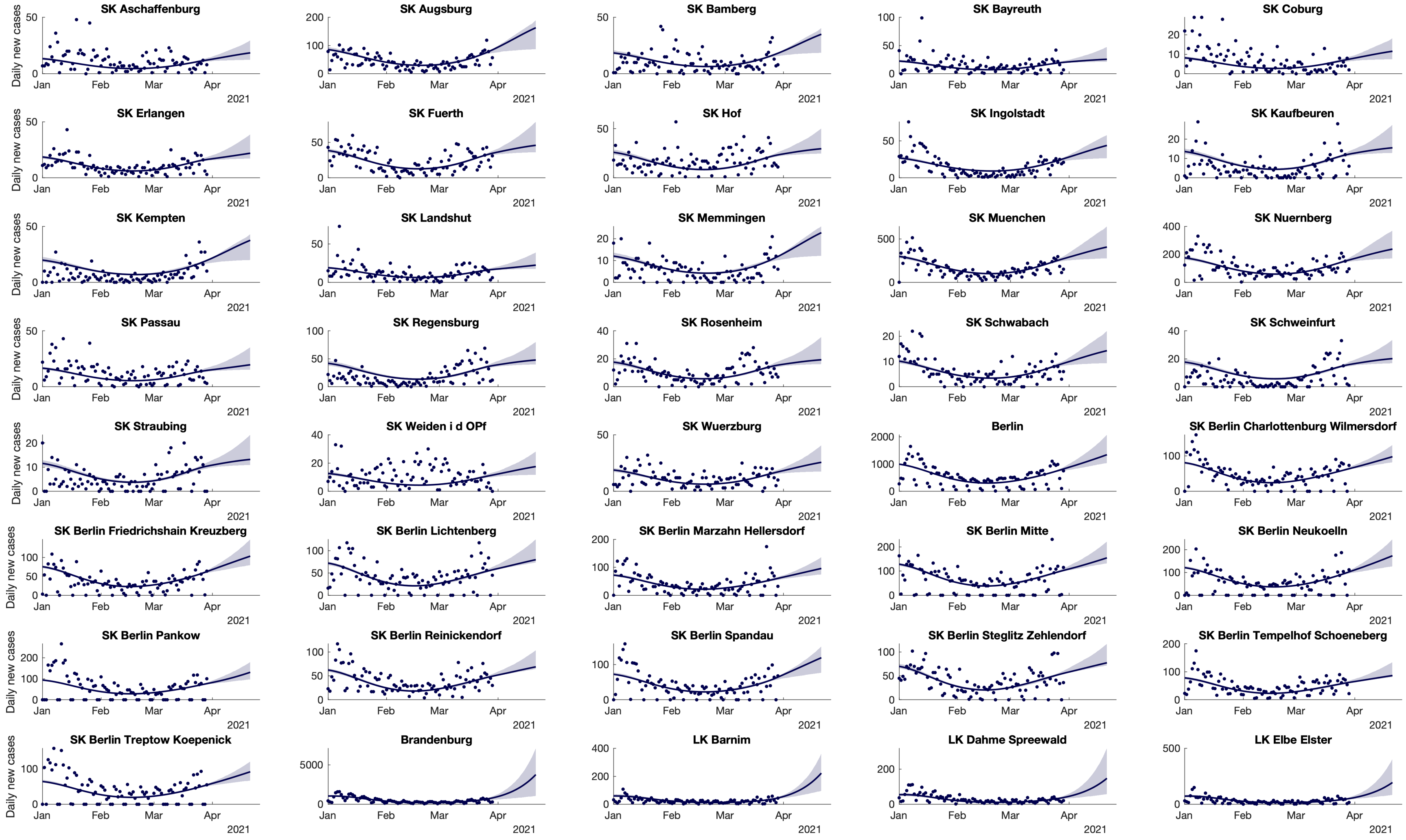
SK Aschaffenburg
SK Augsburg
SK Bamberg
SK Bayreuth
SK Coburg
SK Erlangen
SK Fuerth
SK Hof
SK Ingolstadt
SK Kaufbeuren
SK Kempten
SK Landshut
SK Memmingen
SK Muenchen
SK Nuernberg
SK Passau
SK Regensburg
SK Rosenheim
SK Schwabach
SK Schweinfurt
SK Straubing
SK Weiden i d OPf
SK Wuerzburg
Berlin
SK Berlin Charlottenburg Wilmersdorf
SK Berlin Friedrichshain Kreuzberg
SK Berlin Lichtenberg
SK Berlin Marzahn Hellersdorf
SK Berlin Mitte
SK Berlin Neukoelln
SK Berlin Pankow
SK Berlin Reinickendorf
SK Berlin Spandau
SK Berlin Steglitz Zehlendorf
SK Berlin Tempelhof Schoeneberg
SK Berlin Treptow Koepenick
Brandenburg
LK Barnim
LK Dahme Spreewald
LK Elbe Elster
Daily new cases
Jan
Feb
Mar
Apr
2021

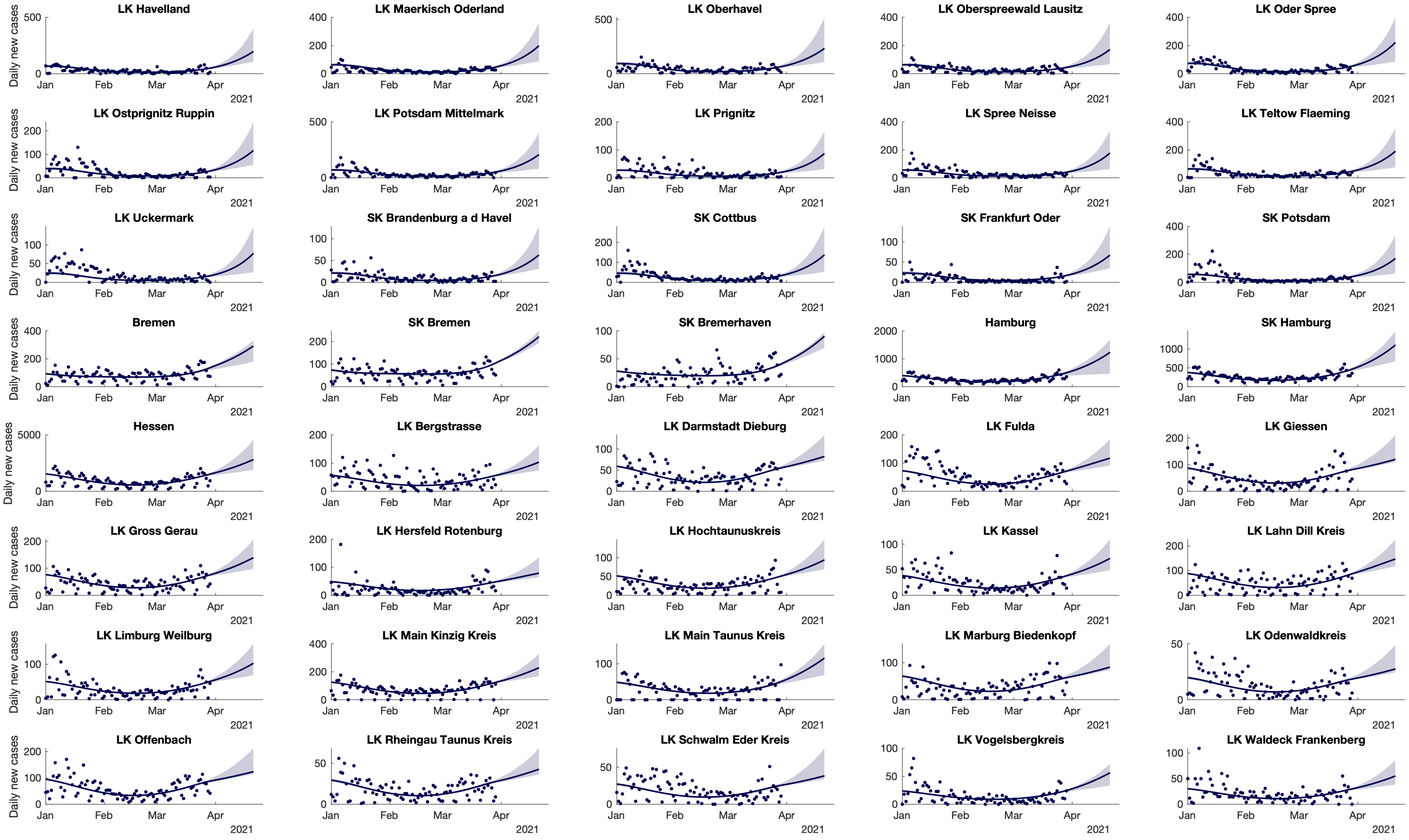
LK Havelland
LK Maerkisch Oderland
LK Oberhavel
LK Oberspreewald Lausitz
LK Oder Spree
LK Ostprignitz Ruppin
LK Potsdam Mittelmark
LK Prignitz
LK Spree Neisse
LK Teltow Flaeming
LK Uckermark
SK Brandenburg a d Havel
SK Cottbus
SK Frankfurt Oder
SK Potsdam
Bremen
SK Bremen
SK Bremerhaven
Hamburg
SK Hamburg
Hessen
LK Bergstrasse
LK Darmstadt Dieburg
LK Fulda
LK Giessen
LK Gross Gerau
LK Hersfeld Rotenburg
LK Hochtaunuskreis
LK Kassel
LK Lahn Dill Kreis
LK Limburg Weilburg
LK Main Kinzig Kreis
LK Main Taunus Kreis
LK Marburg Biedenkopf
LK Odenwaldkreis
LK Offenbach
LK Rheingau Taunus Kreis
LK Schwalm Eder Kreis
LK Vogelsbergkreis
LK Waldeck Frankenberg
Daily new cases
Jan
Feb
Mar
Apr
2021

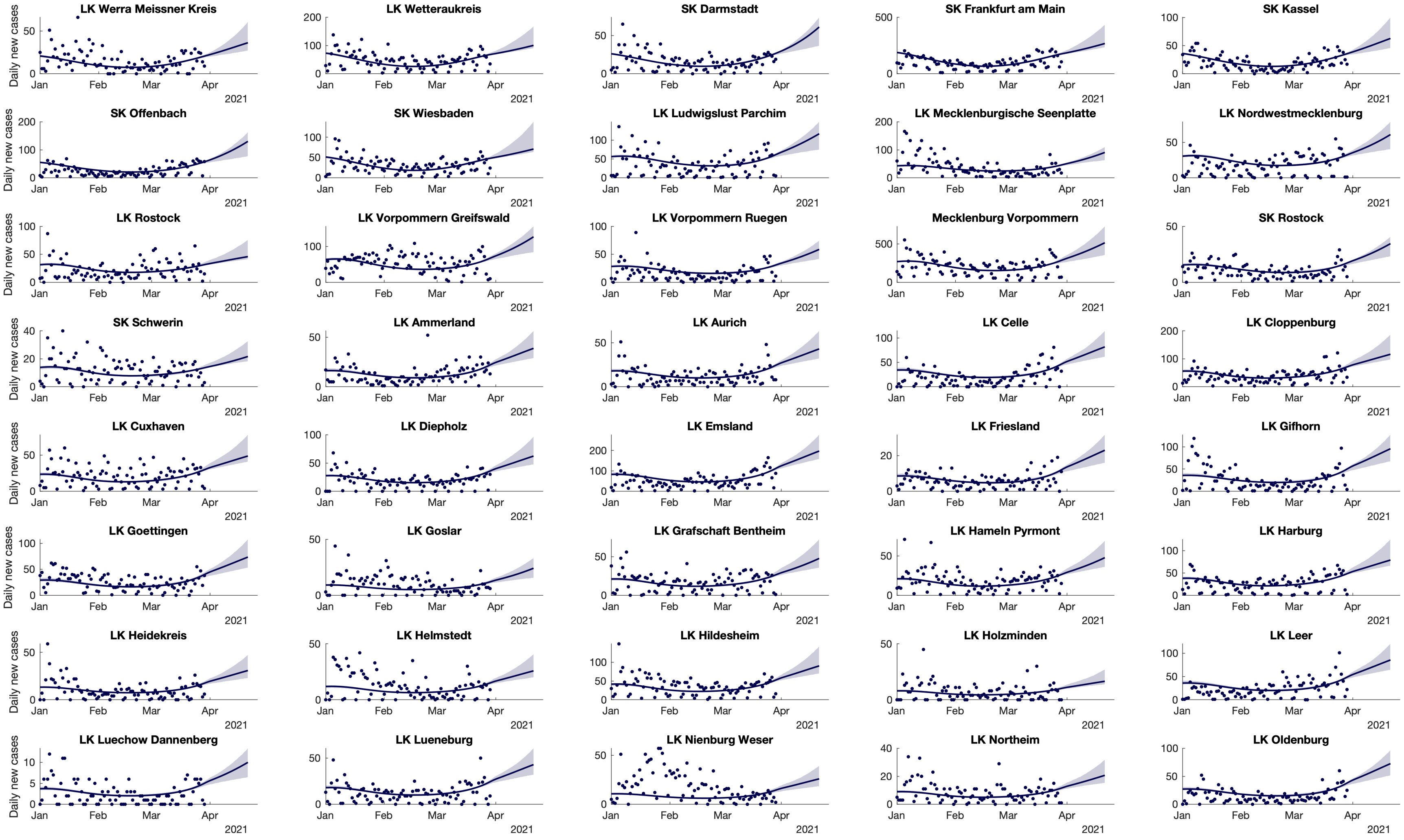
LK Werra Meissner Kreis
LK Wetteraukreis
SK Darmstadt
SK Frankfurt am Main
SK Kassel
SK Offenbach
SK Wiesbaden
LK Ludwigslust Parchim
LK Mecklenburgische Seenplatte
LK Nordwestmecklenburg
LK Rostock
LK Vorpommern Greifswald
LK Vorpommern Ruegen
Mecklenburg Vorpommern
SK Rostock
SK Schwerin
LK Ammerland
LK Aurich
LK Celle
LK Cloppenburg
LK Cuxhaven
LK Diepholz
LK Emsland
LK Friesland
LK Gifhorn
LK Goettingen
LK Goslar
LK Grafschaft Bentheim
LK Hameln Pyrmont
LK Harburg
LK Heidekreis
LK Helmstedt
LK Hildesheim
LK Holzminden
LK Leer
LK Luechow Dannenberg
LK Lueneburg
LK Nienburg Weser
LK Northeim
LK Oldenburg
Daily new cases
Jan
Feb
Mar
Apr
2021

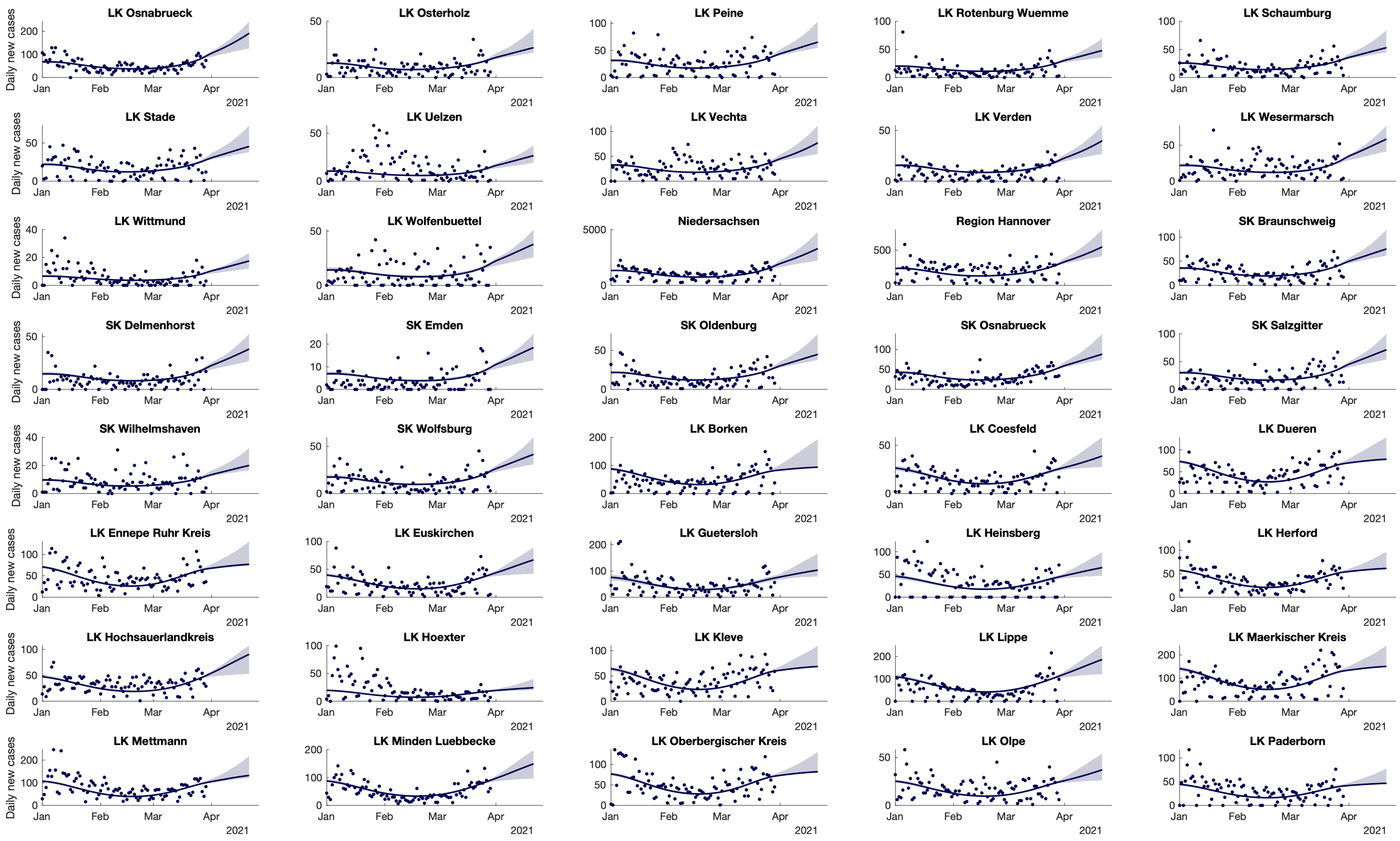

Daily new cases
LK Osnabrueck
LK Osterholz
LK Peine
LK Rotenburg Wuemme
LK Schaumburg
LK Stade
LK Uelzen
LK Vechta
LK Verden
LK Wesermarsch
LK Wittmund
LK Wolfenbuettel
Niedersachsen
Region Hannover
SK Braunschweig
SK Delmenhorst
SK Emden
SK Oldenburg
SK Osnabrueck
SK Salzgitter
SK Wilhelmshaven
SK Wolfsburg
LK Borken
LK Coesfeld
LK Dueren
LK Ennepe Ruhr Kreis
LK Euskirchen
LK Guetersloh
LK Heinsberg
LK Herford
LK Hochsauerlandkreis
LK Hoexter
LK Kleve
LK Lippe
LK Maerkischer Kreis
LK Mettmann
LK Minden Luebbecke
LK Oberbergischer Kreis
LK Olpe
LK Paderborn
Jan
Feb
Mar
Apr
2021

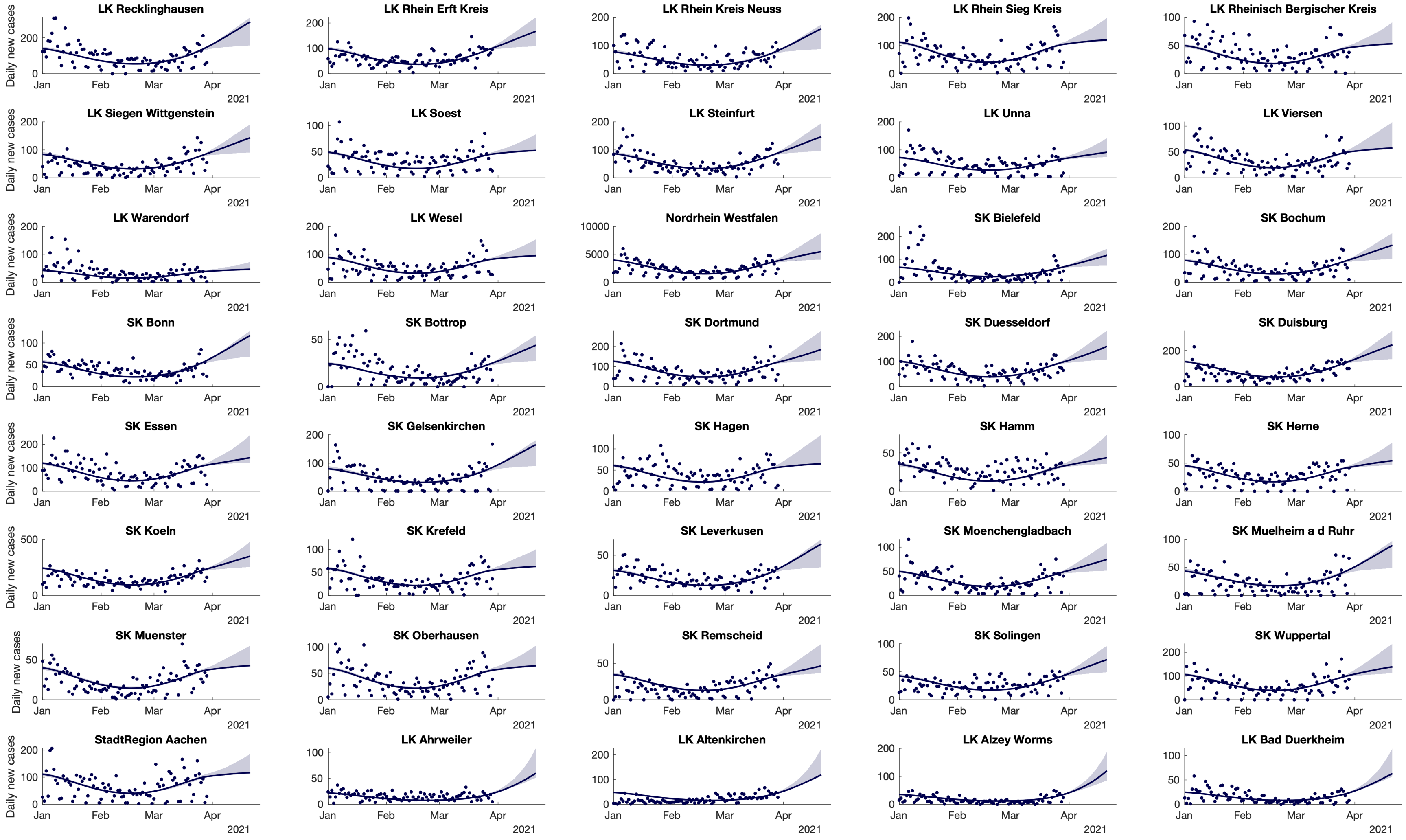
LK Recklinghausen
LK Rhein Erft Kreis
LK Rhein Kreis Neuss
LK Rhein Sieg Kreis
LK Rheinisch Bergischer Kreis
LK Siegen Wittgenstein
LK Soest
LK Steinfurt
LK Unna
LK Viersen
LK Warendorf
LK Wesel
Nordrhein Westfalen
SK Bielefeld
SK Bochum
SK Bonn
SK Bottrop
SK Dortmund
SK Duesseldorf
SK Duisburg
SK Essen
SK Gelsenkirchen
SK Hagen
SK Hamm
SK Herne
SK Koeln
SK Krefeld
SK Leverkusen
SK Moenchengladbach
SK Muelheim a d Ruhr
SK Muenster
SK Oberhausen
SK Remscheid
SK Solingen
SK Wuppertal
StadtRegion Aachen
LK Ahrweiler
LK Altenkirchen
LK Alzey Worms
LK Bad Duerkheim
Daily new cases
Jan
Feb
Mar
Apr
2021

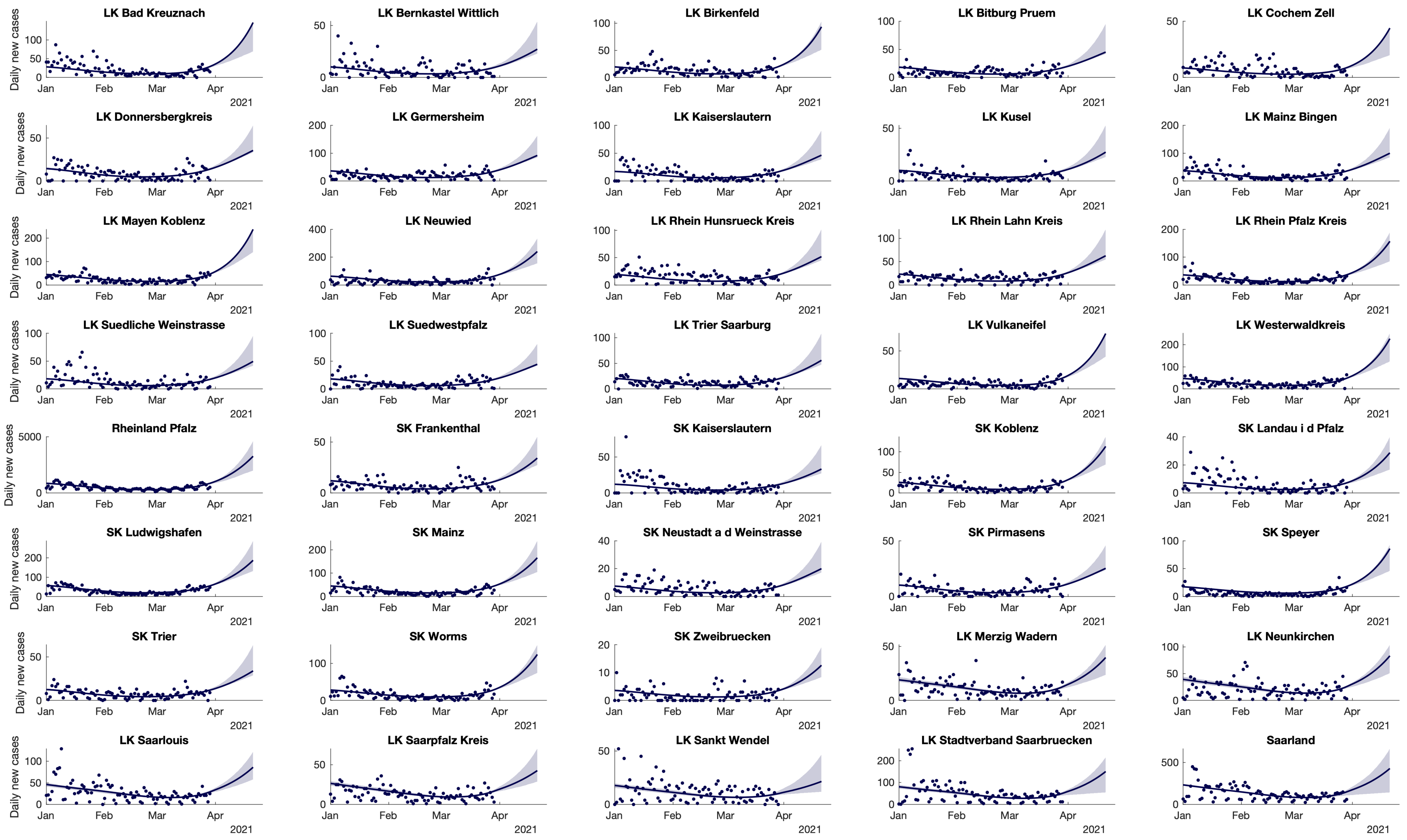
LK Bad Kreuznach
LK Bernkastel Wittlich
LK Birkenfeld
LK Bitburg Pruem
LK Cochem Zell
LK Donnersbergkreis
LK Germersheim
LK Kaiserslautern
LK Kusel
LK Mainz Bingen
LK Mayen Koblenz
LK Neuwied
LK Rhein Hunsrueck Kreis
LK Rhein Lahn Kreis
LK Rhein Pfalz Kreis
LK Suedliche Weinstrasse
LK Suedwestpfalz
LK Trier Saarburg
LK Vulkaneifel
LK Westerwaldkreis
Rheinland Pfalz
SK Frankenthal
SK Kaiserslautern
SK Koblenz
SK Landau i d Pfalz
SK Ludwigshafen
SK Mainz
SK Neustadt a d Weinstrasse
SK Pirmasens
SK Speyer
SK Trier
SK Worms
SK Zweibruecken
LK Merzig Wadern
LK Neunkirchen
LK Saarlouis
LK Saarpfalz Kreis
LK Sankt Wendel
LK Stadtverband Saarbruecken
Saarland
Daily new cases
Jan
Feb
Mar
Apr
2021

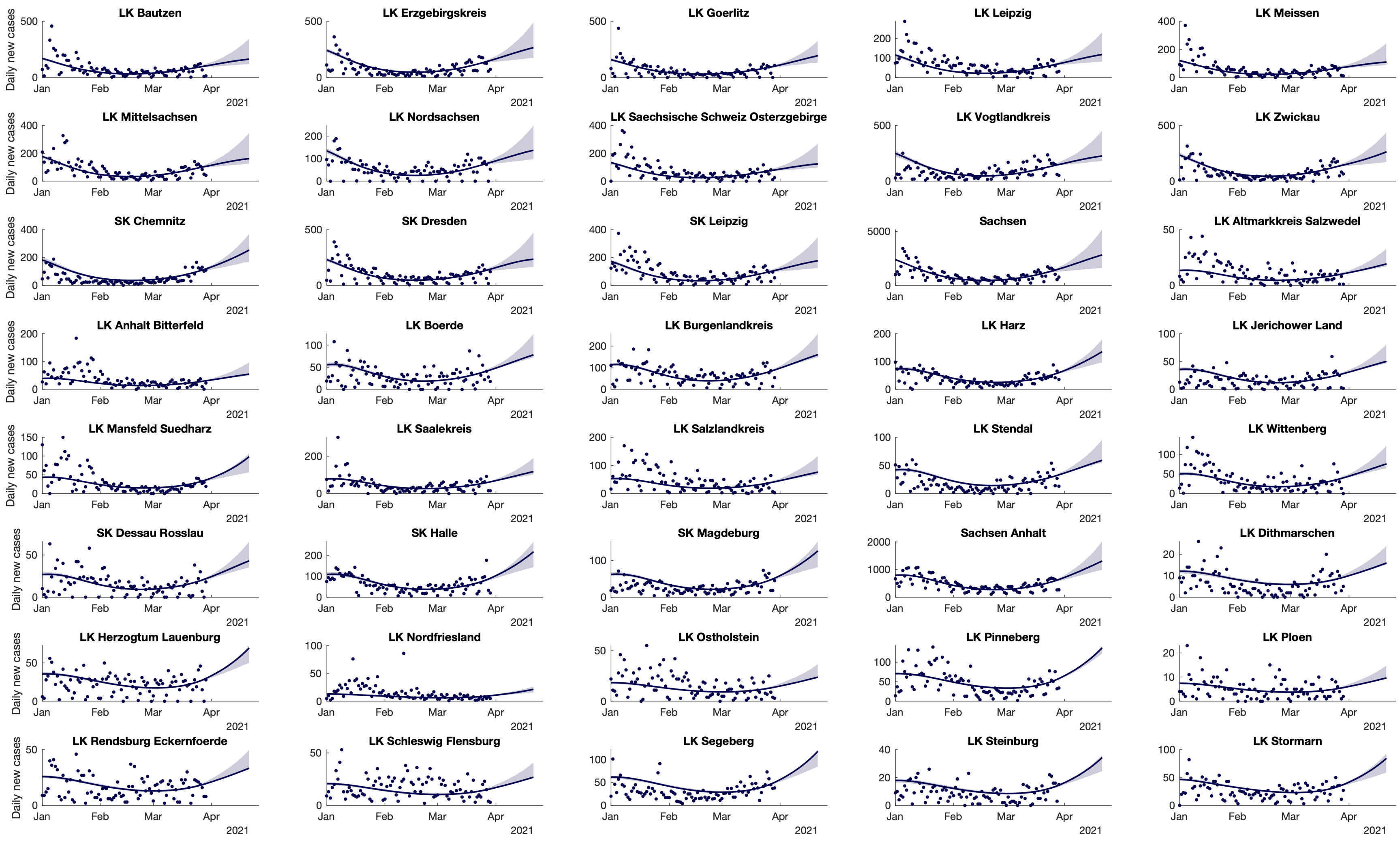

LK Bautzen
LK Erzgebirgskreis
LK Goerlitz
LK Leipzig
LK Meissen
LK Mittelsachsen
LK Nordsachsen
LK Saechsische Schweiz Osterzgebirge
LK Vogtlandkreis
LK Zwickau
SK Chemnitz
SK Dresden
SK Leipzig
Sachsen
LK Altmarkkreis Salzwedel
LK Anhalt Bitterfeld
LK Boerde
LK Burgenlandkreis
LK Harz
LK Jerichower Land
LK Mansfeld Suedharz
LK Saalekreis
LK Salzlandkreis
LK Stendal
LK Wittenberg
SK Dessau Rosslau
SK Halle
SK Magdeburg
Sachsen Anhalt
LK Dithmarschen
LK Herzogtum Lauenburg
LK Nordfriesland
LK Ostholstein
LK Pinneberg
LK Ploen
LK Rendsburg Eckernfoerde
LK Schleswig Flensburg
LK Segeberg
LK Steinburg
LK Stormarn
Daily new cases
Jan
Feb
Mar
Apr
2021

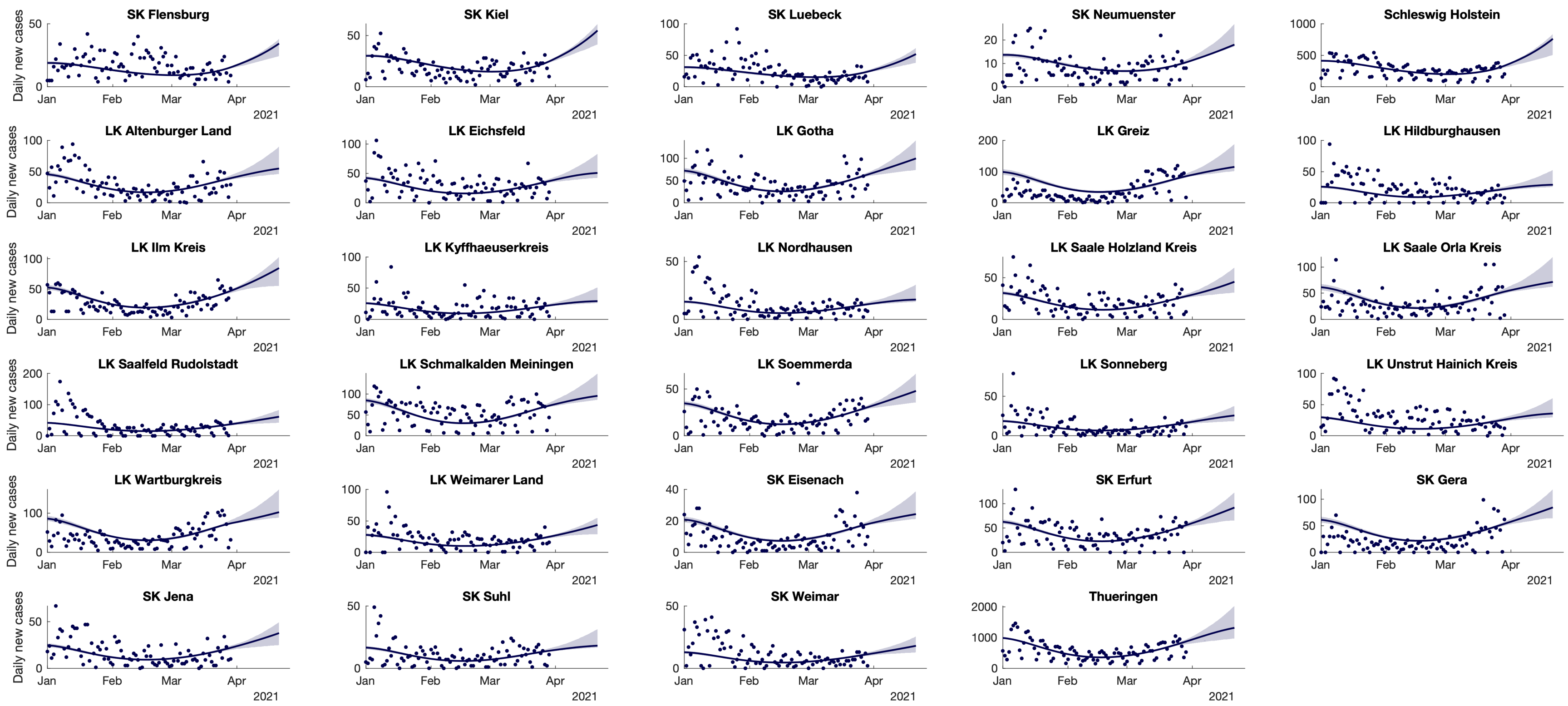
SK Flensburg
SK Kiel
SK Luebeck
SK Neumuenster
Schleswig Holstein
LK Altenburger Land
LK Eichsfeld
LK Gotha
LK Greiz
LK Hildburghausen
LK Ilm Kreis
LK Kyffhaeuserkreis
LK Nordhausen
LK Saale Holzland Kreis
LK Saale Orla Kreis
LK Saalfeld Rudolstadt
LK Schmalkalden Meiningen
LK Soemmerda
LK Sonneberg
LK Unstrut Hainich Kreis
LK Wartburgkreis
LK Weimarer Land
SK Eisenach
SK Erfurt
SK Gera
SK Jena
SK Suhl
SK Weimar
Thueringen
Daily new cases
Jan
Feb
Mar
Apr
2021
